# Keratinocytes and PDGF-B/PDGFRβ signaling modulate peripheral opioid tolerance

**DOI:** 10.1016/j.isci.2026.116634

**Published:** 2026-06-30

**Authors:** Luca Posa, Angelique J. Buton, Anita M. Khasnavis, Sophia A. Miracle, Kathryn M. Albers, Matthew J. Fanelli, Timmy Le, Mackenzie Gamble, Ashley K. McDonald, Gilles Martin, Salome Fabri-Ruiz, Zachary Freyberg, Ryan W. Logan, Stephanie Puig

**Affiliations:** 1 Department of Pharmacology, Physiology and Biophysics, Boston University Chobanian and Avedisian School of Medicine, Boston, MA 02118, USA; 2 Department of Psychiatry and Behavioral Sciences, University of Massachusetts Chan Medical School, Worcester, MA 01605, USA; 3 Department of Neurobiology, University of Massachusetts Chan Medical School, Worcester, MA 01605, USA; 4 Brudnick Neuropsychiatric Research Institute, University of Massachusetts Chan Medical School, Worcester, MA 01605, USA; 5 Graduate Program of Neuroscience, Morningside Graduate School of Biological Sciences, University of Massachusetts Chan Medical School, Worcester, MA 01605, USA; 6 Department of Neurobiology, University of Pittsburgh, Pittsburgh, PA, USA; 7 Molecular and Translational Medicine, Department of Medicine, Boston University Chobanian and Avedisian School of Medicine, Boston, MA, USA; 8 DECOD, L’Institut Agro, IFREMER, INRAE, Nantes 44311, France; 9 Department of Psychiatry, University of Pittsburgh, Pittsburgh, PA 15213, USA; 10 Department of Cell Biology, University of Pittsburgh, Pittsburgh, PA 15213, USA

**Keywords:** opioid analgesia, Peripheral opioid tolerance, Keratinocytes, growth factors

## Abstract

Peripheral opioids are safer than systemic opioids by avoiding central side effects. However, peripheral tolerance hampers their clinical use. We highlight an epithelial-neuronal communication mechanism for peripheral opioid tolerance. We discovered that both repeated intraplantar (i.pl.) morphine injections or repeated keratinocyte photostimulation in mice cause peripheral tolerance in a platelet-derived growth factor type B (PDGF-B) and PDGF receptor beta (PDGFRβ)-dependent manner. We also find that morphine i.pl. increases PDGF-B in mu-opioid receptor-expressing keratinocytes and alters the electrophysiological properties of keratinocytes. This underscores the importance of keratinocytes and the necessity for PDGF-B release for peripheral tolerance, though the source of PDGF-B remains undetermined. Nevertheless, we show that PDGFRβ inhibition completely blocks peripheral tolerance in peripheral chronic pain models, highlighting the translational relevance of our findings. Overall, we highlight that keratinocytes and PDGFRβ are promising targets to reduce peripheral tolerance, which could allow shifting from systemic to peripheral opioid delivery and increase opioid safety.

## Highlights

• PDGF-B/PDGFRβ are necessary and sufficient for peripheral morphine tolerance
• Moprhine i.pl. increases PDGF-B in keratinocytes and alters their electrophysiology
• Keratinocyte photostimulation causes tolerance blocked by PDGF-B/PDGFRβ inhibition
• PDGFRβ inhibitor blocks peripheral morphine tolerance in models of chronic pain

## Graphical abstract

**Figure:**
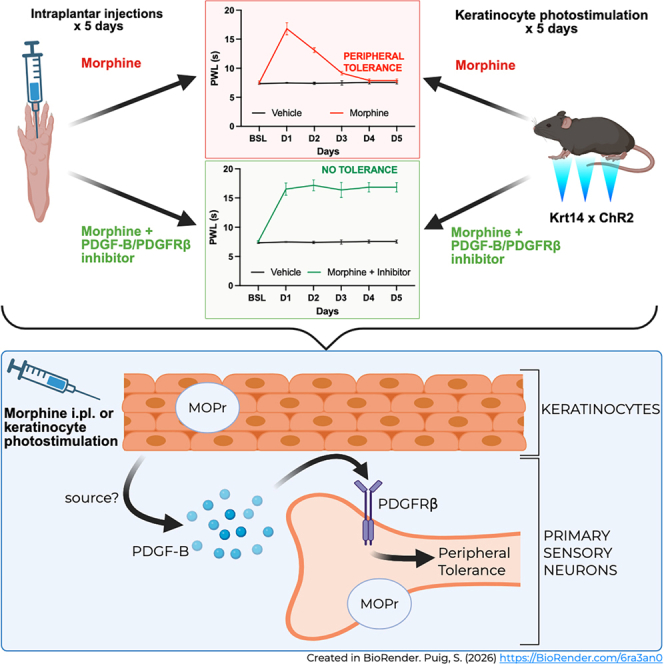

## Teaser

Molecular biology; Neuroscience; Pharmacology

## Introduction

Opioids are powerful analgesic compounds that have long served as a cornerstone treatment for severe pain.^1^ However, repeated opioid use leads to tolerance, which requires escalating doses to overcome the reduction of analgesia over time. Dose-escalation reduces opioid safety by increasing deleterious centrally mediated side effects such as physical dependence, respiratory depression, and/or addiction, which can lead to overdose and death. Innovative strategies to increase opioid safety without affecting analgesia are needed. Clinical evidence shows that peripheral and topical application of low doses of opioids produces effective analgesia, while limiting central penetration^2^^,^^3^^,^^4^^,^^5^ and adverse side effects.^3^^,^^6^^,^^7^^,^^8^^,^^9^ Peripheral and topical opioid formulations are in phase III clinical trials,^10^^,^^11^ yet specific mechanisms of action for peripheral opioids remain poorly understood.^12^^,^^13^^,^^14^ In addition, even with peripherally administered opioids, tolerance to analgesia develops,^14^^,^^15^^,^^16^^,^^17^^,^^18^ overshadowing their clinical utility.^2^^,^^5^^,^^19^^,^^20^

Opioids drive analgesia via μ-opioid receptor (MOPr) signaling.^21^ Centrally, opioid activation of MOPr causes spinal release of platelet-derived growth factor B (PDGF-B), which activates PDGF receptor beta (PDGFRβ) to directly cause spinal opioid tolerance.^22^ Accordingly, imatinib, a PDGFRβ inhibitor, completely blocks spinal opioid tolerance,^22^ making PDGFRβ a promising target to prevent CNS tolerance. We therefore investigated whether PDGFRβ could also be an effective target to prevent peripheral opioid tolerance. In the periphery, MOPr, PDGFRβ, and the PDGF-B ligand are expressed in somas of primary sensory neurons (PSNs) of dorsal root ganglia (DRG).^23^^,^^24^^,^^25^^,^^26^^,^^27^^,^^28^^,^^29^ There is also evidence for MOPr and PDGFRβ proteins in PSN nerve endings in the skin.^26^ Skin keratinocytes similarly express MOPr^25^^,^^30^^,^^31^^,^^32^^,^^33^ and PDGF-B.^34^^,^^35^^,^^36^^,^^37^^,^^38^ Skin MOPr activation impacts wound healing, inflammation, and itch,^31^^,^^32^^,^^39^ but its role in tolerance remains unclear.^40^^,^^41^ The convergence of MOPr and PDGF-B/PDGFR-β localization in peripheral tissue led us to hypothesize that PDGF-B/PDGFR-β signaling has a role in peripheral opioid tolerance.^42^ In addition, the direct involvement of keratinocytes in somatosensation^43^^,^^44^^,^^45^^,^^46^ raises the possibility that they are a component of the circuitry mediating peripheral tolerance. Anatomical and functional evidence indicate that keratinocytes communicate with PSN endings to modulate somatosensory information through keratinocyte-derived factors.^43^^,^^45^^,^^47^^,^^48^^,^^49^^,^^50^

In this study, we used behavioral and pharmacological approaches to examine the role of PDGF-B/PDGFR-β signaling in peripheral opioid tolerance caused by repeated intraplantar (i.pl.) morphine administration in mice. The impact of i.pl. morphine administration and keratinocyte stimulation on the expression of MOPr and PDGF-B in keratinocytes were examined using RNAscope fluorescent *in situ* hybridization. Patch-clamp electrophysiological recording on primary cultures of keratinocytes was used to examine the impact of repeated peripheral morphine administration on keratinocytes' biophysical properties. Finally, targeted optogenetics was also used to determine the effect of keratinocyte photostimulation on the development of peripheral morphine analgesia and tolerance. We discovered that morphine peripheral tolerance is blocked by the selective inhibition of PDGFRβ or PDGF-B in the periphery. Data also revealed that repeated, but not acute, i.pl. PDGF-B induced peripheral morphine tolerance. Thus, repeated peripheral stimulation of PDGF-B/PDGFRβ signaling is necessary and sufficient to cause peripheral morphine tolerance. Because our prior and current findings show that PDGFRβ is expressed in PSNs but not in keratinocytes, and repeated but not acute morphine i.pl. causes PDGFRβ phosphorylation in PSNs, we propose that the inhibition of peripheral tolerance with PDGF-B/PDGFRβ inhibitors occurs via inhibition of morphine-mediated activation of PDGFRβ in PSNs. In addition, repeated keratinocyte photostimulation is also sufficient to cause a peripheral tolerance-like state where morphine tolerance is accompanied by the alteration of biophysical properties of keratinocytes as measured by patch-clamp electrophysiology. Finally, peripheral co-administration of morphine and imatinib efficiently blocks peripheral tolerance and potentiates morphine analgesic efficacy in a model of peripheral inflammatory pain and in a model of post-surgical pain.

Overall, our study shows that peripheral morphine tolerance involves PDGF-B/PDGFRβ-dependent signaling mechanisms. Additionally, keratinocytes may be critical in the etiology of peripheral tolerance. Therefore, epithelial-neural communication and activation of PDGF-B/PDGFRβ signaling lead to peripheral tolerance. Most importantly, this study reveals that PDGFRβ is a promising target for managing the effective treatment of peripheral chronic pain conditions.

## Results

### Inhibition of PDGFRβ prevents peripheral morphine tolerance

**Figure 1:**
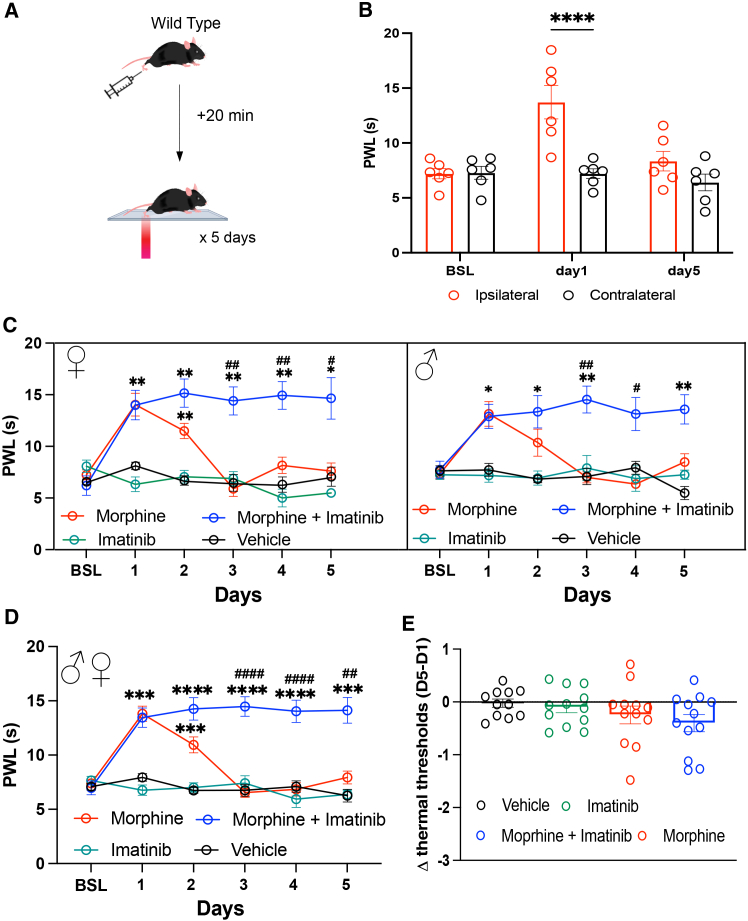
Imatinib blocks peripheral morphine tolerance (A) Schematic of the experimental design. (B) Intraplantar (i.pl.) morphine administration induces ipsilateral but not contralateral acute antinociception as seen by an increase in paw withdrawal latency (PWL) on day 1. Tolerance develops by day 5. See also Figures S1A and S1B. (C) Morphine peripheral tolerance develops upon repeated morphine i.pl. injections. Co-injection of morphine + imatinib blocks peripheral tolerance in female (C left) and male (C right) mice. (D) Females and males’ data merged show that the combined injection of morphine + imatinib can block peripheral morphine tolerance in a sex-independent manner. See also Figure S1C. (E) Repeated i.pl. administrations of morphine do not induce thermal hypersensitivity in the ipsilateral paw. For all figures: BSL = baseline, D = day. MS = Morphine Sulfate, *N* = 6 per group/sex. ∗*p* < 0.05, ∗∗*p* < 0.01, ∗∗∗*p* < 0.001, and ∗∗∗∗*p* < 0.0001 vs. vehicle, #*p* < 0.05 ##*p* < 0.01, and ####*p* < 0.0001 vs. morphine. Two-way or three-way Repeated Measures ANOVA followed by Šídák’s (B) or Tukey’s (C and D) multiple comparisons test. Data are expressed as mean ± S.E.M. Detailed statistics information can be found in Table S1. See supplemental information in Figure S1.

We first established a model of peripheral morphine tolerance (Figures 1A, S1A, and S1B). We conducted a dose-response study to determine the intraplantar (i.pl.) dose of morphine necessary to induce local ipsilateral but not contralateral analgesia (Figures S1A and S1B, *N* = 6, two-way ANOVA, dose x treatment: F (4, 40) = 341.2, *p* < 0.0001). A dose of 5 μg per injection was selected and was confirmed to induce ipsilateral analgesia on day 1 and tolerance by day 5 (*N* = 6 mice/group, two-way ANOVA, time x paw, F (2, 20) = 8.954, *p* = 0.0017, Figure 1B and Table S1). To test the impact of the PDGFRβ inhibitor, imatinib, on peripheral morphine tolerance, female (Figure 1C left) and male (Figure 1C right) mice received either vehicle (Veh) (5 μL), morphine alone (5μg/5 μL), imatinib alone (10μg/5 μL), or the combination of morphine + imatinib i.pl. for 5 consecutive days. Paw withdrawal latencies (PWLs) were measured 20 min later to test thermal analgesia and the development of peripheral tolerance. Groups receiving morphine only developed peripheral morphine tolerance as shown by PWLs returning to baseline levels after 3 days of injections in both sexes (two-way ANOVA Figure 1C and Table S1 *left*: females, *N* = 6/group, time x treatment, F (15, 100) = 9.411, *p* < 0.0001; Figure 1D *right*: males, *N* = 6/group, time x treatment, F (15, 100) = 4.917, *p* < 0.0001). Co-administration of morphine + imatinib i.pl. completely prevented tolerance, while it did not alter analgesia levels on day 1. Thus, demonstrating that imatinib inhibition is sufficient to block peripheral morphine tolerance without affecting acute analgesia. A 3-way ANOVA analysis comparing sex x treatment x time showed no interaction (F (15, 246) = 0.808, *p* = 0.669), revealing no differential sex effect of imatinib on peripheral tolerance (Figure 1C and Table S1). We then combined data from both females and males and confirmed that imatinib prevented morphine tolerance (two-way ANOVA Figure 1D and Table S1: *N* = 12/group, time x treatment, F (15, 220) = 13.22, *p* < 0.0001). This effect was also confirmed for mechanical analgesia and tolerance (Figure S1C: *N* = 3/sex/group, time x treatment, F (2.868, 14.34) = 8.784, *p* = 0.0016). Finally, 5 days of repeated morphine i.pl. injections did not cause thermal or mechanical opioid induced hyperalgesia (OIH) (1-way ANOVA, thermal OIH: Figure 1E, N = 5–6/sex/group, F (3, 43) = 1.482, *p* = 0.2328, mechanical OIH: Figure S1D, *N* = 3/sex/group, F (3, 20) = 0.5123, *p* = 0.6784, Table S1). Overall, we show that imatinib is sufficient to completely block peripheral morphine tolerance.

### Selective anti-PDGFRβ mAb blocks peripheral morphine tolerance

**Figure 2:**
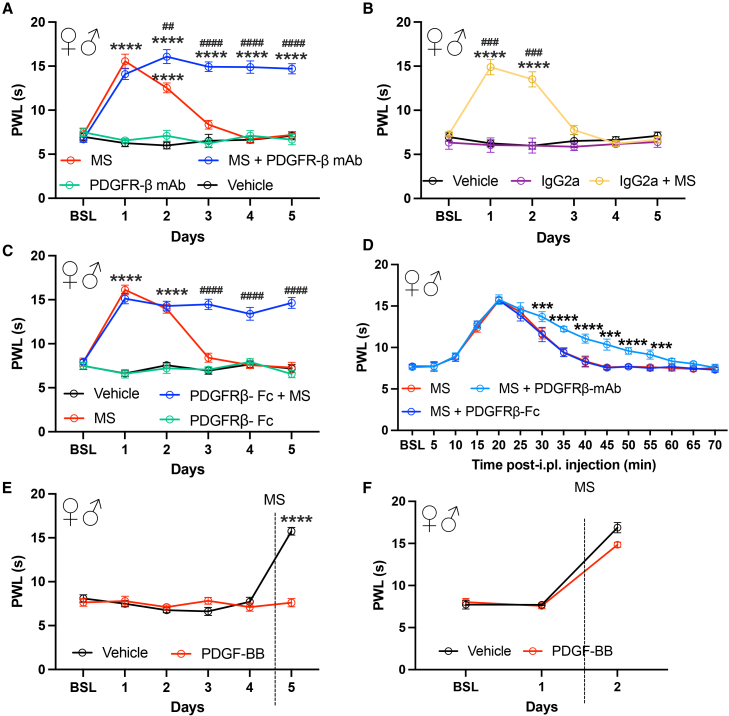
PDGFRβ signaling is both necessary and sufficient for peripheral morphine tolerance (A) Repeated co-injection of morphine + anti-PDGFRβ monoclonal antibody (mAb) blocks peripheral tolerance. Two-way Repeated Measures ANOVA followed by Tukey’s multiple comparisons test. BSL = baseline, N = 4–5 per group/sex. ∗∗∗∗*p* < 0.0001 vs. vehicle, ##*p* < 0.01, ####*p* < 0.0001 vs. morphine. See also the separated female and male data in Figure S2A. (B) Control experiment. Neutral IgG2a mAb does not affect morphine acute analgesia on day 1 and does not prevent the development of tolerance overtime. Two-way Repeated Measures ANOVA followed by Tukey’s multiple comparisons test. BSL = baseline, N = 8–10 per group/sex. ∗∗∗∗*p* < 0.0001 vs. vehicle, ###*p* < 0.001 vs. morphine. (C) Repeated co-injection of morphine + the PDGF-B scavenger, PDGFRβ Fc, blocks peripheral tolerance. Two-way Repeated Measures ANOVA followed by Tukey’s multiple comparisons test. MS = Morphine Sulfate, BSL = baseline, *N* = 3 per group/sex. ∗∗∗∗*p* < 0.0001 vs. vehicle, ####*p* < 0.0001 vs. morphine. See also the separated female and male data in Figure S2B. (D) Acute co-injection of morphine + anti-PDGFRβ mAb prolongs morphine peripheral analgesia, while acute co-injection of morphine + PDGFRβ -Fc does not change the time course of acute morphine analgesia. Two-way Repeated Measures ANOVA followed by Dunnett’s multiple comparisons test. BSL = baseline, *N* = 3 per group/sex. ∗∗∗*p* < 0.001 and ∗∗∗∗*p* < 0.0001 vs. morphine. (E) Repeated i.pl. administration of recombinant PDGF-B blocks analgesia to a morphine challenge on day 5. Two-way repeated measures ANOVA followed by Dunnett’s multiple comparisons test. BSL = baseline, *N* = 5–6 per group/sex. ∗∗∗∗*p* < 0.0001 vs. vehicle BSL. See also the separated female and male data in Figure S2C. (F) A single intraplantar administration of recombinant PDGF-B does not alter analgesia to a morphine challenge the following day. Two-way Repeated Measures ANOVA followed by Dunnett’s multiple comparisons test. *N* = 3 per group/sex. ∗∗∗∗*p* < 0.0001 vs. vehicle BSL, ####*p* < 0.0001 vs. PDGF-B BSL. For all figures: Data are expressed as mean ± s.e.m. Detailed statistics information can be found in Table S2. See supplemental information in Figures S2 and S3.

Though imatinib is a well-established PDGFRβ inhibitor, it also has many other known receptor tyrosine kinase targets.^51^^,^^52^^,^^53^^,^^54^^,^^55^^,^^56^^,^^57^ Therefore, to thoroughly examine the specificity of PDGFRβ inhibition on peripheral tolerance, we used a neutralizing monoclonal antibody (mAb), anti-PDGFRβ. When morphine was co-administered with PDGFRβ mAb for five days, peripheral morphine tolerance was completely attenuated, confirming that PDGFRβ mAb blocked morphine tolerance (two-way ANOVA Figure 2A and Table S2: N = 9–10 mice per group, time x treatment, F (13.59, 145.0) = 22.82, *p* < 0.0001). Analysis of female and male mice data separately did not show any sex effect (N = 4–5 group, two-way ANOVA, Figure S2A *left*: females, time x treatment, F (15, 70) = 9.797, *p* < 0.0001; Figure S2A *right*: males, time x treatment, F (15, 70) = 16.33, *p* < 0.0001 Table S2). No sex difference was confirmed by 3-way ANOVA analysis (N = 4–5/sex/treatment, F (15, 168) = 1.376, *p* = 0.164, Table S2). Importantly, no change in thermal nociceptive threshold was observed over time in the groups treated with the mAb alone, showing that: (1) mAb PDGFRβ selective inhibition does not induce analgesia, and (2) the effect observed in combination with morphine specifically blocks peripheral morphine tolerance. To further confirm that the i.pl. presence of an IgG2a isotype was not mediating these effects, so we controlled for the absence of effect of IgG2a injection on morphine analgesia and tolerance. We co-administered morphine i.pl. with IgG2a kappa isotype control antibody and found that it did not affect peripheral morphine acute analgesia and development of peripheral tolerance (Figure 2B, N = 4–9/group, two-way ANOVA, time x treatment, F (7.647, 68.82) = 19.22, *p* < 0.0001, Table S2), thus confirming that the effect of PDGFRβ-mAb on peripheral morphine tolerance is due to its action on PDGFRβ, not due to the i.pl. injection of an IgG2a mAb. Collectively, these results support a specific role for PDGFR-β in mediating peripheral morphine tolerance.

### PDGF-B is necessary and sufficient to cause peripheral tolerance

We next examined whether the endogenous PDGFR-β ligand, PDGF-B, is necessary for the establishment of peripheral morphine tolerance. The PDGFR-β-Fc chimera protein (R&D Systems), which consists of a fusion of the N-terminal ligand-binding domain of PDGFR-β with the Fc fragment of an IgG_1_, was used to confer solubility. This construct acts as a scavenger of extracellular PDGF ligands, thereby preventing binding to PDGFRβ. Co-administration of morphine and PDGFRβ-Fc i.pl. completely abolished peripheral morphine tolerance (Figure 2C and two-way ANOVA; Table S2: *N* = 12/group, time x treatment, F (13.43, 196.9) = 28.01, *p* < 0.0001), supporting PDGFRβ-Fc as an effective inhibitor of peripheral morphine tolerance. Analysis of female and male mice data separately indicated similar observations in both females and males (Females: Figure S2B *left*, *N* = 6/group, two-way ANOVA, time x treatment, F (12.15, 81.02) = 14.89, *p* < 0.0001, Table S2; Males: Figure S2B *right*, *N* = 6/group, two-way ANOVA, time x treatment, F (11.88, 79.23) = 13.44, *p* < 0.0001, Table S2). A 3-way ANOVA analysis confirmed no sex difference between females and males (*N* = 6/sex/group: F (15, 240) = 0.55, *p* = 0.907, Table S2). These results show that extracellular PDGF-B at the periphery is necessary to cause peripheral morphine tolerance. Because our daily behavioral testing of peripheral tolerance only assesses the effect of PDGFRβ and PDGF-B inhibition on an acute time point after morphine i.pl. injection (+20 min), we conducted time course experiments to examine the impact of PDGFRβ/PDGF-B inhibition on the acute peak effect and duration of morphine i.pl. analgesia (Figure 2D). Consistent with the results in Figures 2A and 2C, we found that neither PDGFR-β mAb nor PDGFRβ-Fc altered the peak analgesic effect of morphine i.pl. at 20 min. However, we found that PDGFRβ-mAb prolonged the duration of morphine i.pl. analgesia, while PDGFGRβ-Fc did not (Figure 2D: *N* = 6/group, two-way ANOVA, time x treatment, F (10.72, 80.42) = 15.46, *p* < 0.0001, Table S2). These data show that the PDGF ligand is not involved in morphine acute analgesia signaling. However, selective blockade of PDGFRβ signaling promoted longer morphine analgesic effect, suggesting that the activation of PDGFRβ signaling upon morphine administration may have an impact on MOR signaling that causes analgesia.

Next, we determined whether PDGF-B is sufficient to induce peripheral morphine tolerance. Veh or recombinant PDGF-B (rPDGF-B) was administered i.pl. for 4 days and morphine i.pl. was administered on day 5. Veh mice challenged with morphine showed acute antinociception. However, morphine antinociception was completely abolished in mice pre-treated with PDGF-B for 4 days (two-way ANOVA Figure 2E and Table S2: *N* = 11–12/group, time x treatment, F (3.858, 81.01) = 33.17, *p* < 0.0001). Notably, consistent with prior work showing repeated spinal injection of rPDGF-B did not alter baseline thermal thresholds,^22^ repeated i.pl. injection of rPDGF-B did not change PWL thresholds. Analysis of sexes separately did not show a sex effect (Females: Figure S2C *left*, *N* = 6/group, two-way ANOVA, time x treatment, F (3.100, 31.00) = 26.72, *p* < 0.0001, Table S2), Males: Figure S2C *right*, N = 5–6/group, two-way ANOVA, time x treatment, F (3.573, 32.15) = 11.43, Table S2). No interaction of sex x treatment was observed (N = 5–6/sex/treatment, 3-way ANOVA, F (5, 114) = 0.609, *p* = 0.693, Table S2). Interestingly, this effect was not observed after a single i.pl. injection of recombinant PDGF-B (two-way ANOVA, Figure 2F: *N* = 3/sex/group, time x treatment, F (1.544, 15.44) = 21.21, *p* < 0.0001, Table S2). These findings show that morphine i.pl. analgesia is abolished in mice chronically pre-treated with PDGF-B, suggesting that PDGF-B plays a pivotal role in peripheral morphine tolerance, as it is necessary and sufficient to induce peripheral morphine tolerance. Moreover, acute but not chronic i.pl. morphine caused phosphorylation of PDGFRβ in DRGs on tyrosine 1021 (Y1021, Figures S2D and S3, two-way ANOVA N = 3–4/sex/group, time x treatment, F (1, 24) = 2.686, *p* = 0.1143, Table S2). An increase in total PDGFRβ expression was also observed in DRG after chronic morphine i.pl. (Total PDGFRβ, Figures S2E and S3, two-way ANOVA N = 3–4/sex/group, time x treatment, F (1, 26) = 2.154, *p* = 0.1542, Table S2). Thus, chronic morphine leads to the phosphorylation of PDGFRβ in PSNs; PDGFRβ inhibitors may therefore block tolerance by inhibiting morphine-activated PDGFRβ in PSNs.

### Peripheral morphine administration increases PDGF-B expression in keratinocytes

**Figure 3:**
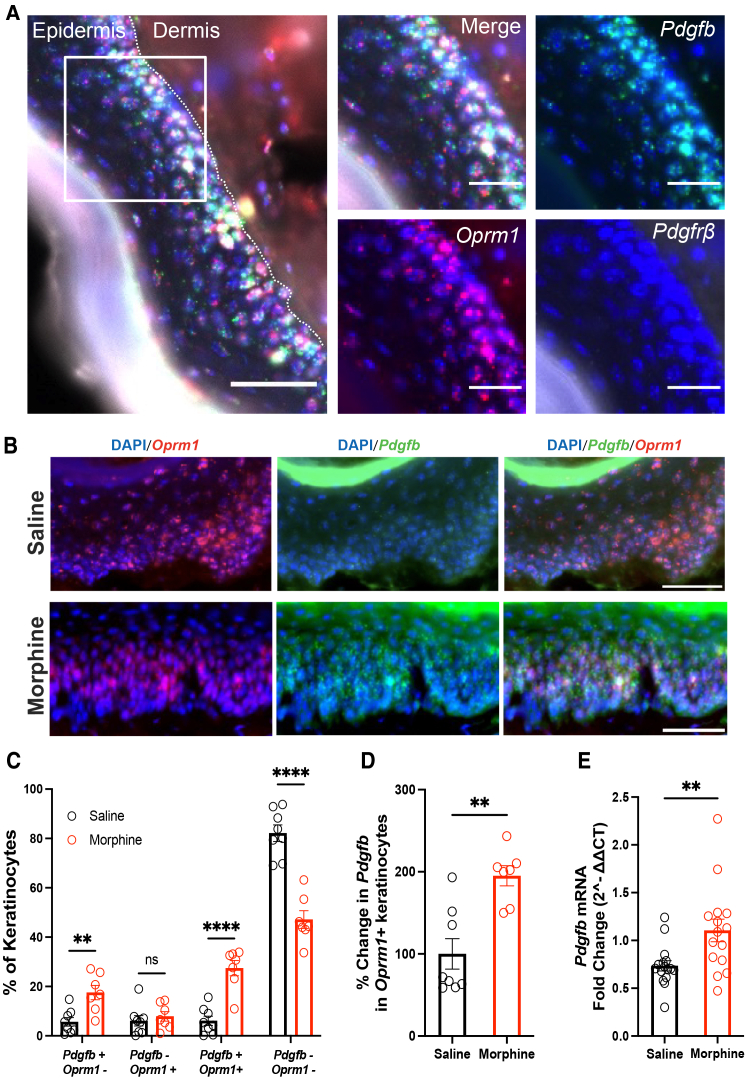
Peripheral morphine administration increases PDGF-B expression in keratinocytes (A) *In situ* hybridization shows co-expression of *Pdgfb* (green) and *Oprm1* (red) mRNA but not *Pdgfrb* (white) mRNA in epidermal keratinocytes. Scale bars: Left = 20 mm, Zoomed-in figures = 10 mm. (B) Representative images of skin from mice injected for five days with saline or morphine i.pl. Scale bars: Left = 20 mm. (C) Quantification of expression highlights that morphine i.pl. for 5 days changes the proportions of keratinocytes expressing *Pdgfb, Oprm1,* or both. “Pdgfb+”: *Pdgfb* expressing cells; “Oprm1+”: *Oprm1* expressing cells; “Pdgfb+Oprm1+”: co-expressing cells; “Pdgfb-Oprm1-”: cells negative for both mRNAs. N = 7–8/group. Two-way ANOVA followed by Sidak’s multiple comparison test. (D) Amount of *Pdgfb* mRNA detected in *Oprm1* cells increases in mice injected with morphine i.pl. for 5 days. N = 7–8/group. Unpaired Student’s t test. (E) Total *Pdgfb* mRNA detected in glabrous skin with RT-PCR does not change after injection with morphine i.pl. for 5 days. *N* = 15-6/group. Unpaired Student’s t test. For all figures: *Pdgfb* = PDGF-B mRNA, *Oprm1* = MOPR mRNA, *Pdgfrb* = PDGFRβ mRNA. ∗∗*p* < 0.01 and ∗∗∗∗*p* < 0.0001, morphine vs. saline/vehicle. Data are expressed as mean ± S.E.M. Detailed statistics information can be found in Table S3.

We previously showed that PDGFR-β is expressed in cutaneous PSNs.^26^ PDGF-B ligand is also present in the periphery, with evidence of expression in epidermal keratinocytes.^34^^,^^35^^,^^36^^,^^37^^,^^38^ MOPr is similarly known to be expressed in DRG neurons^23^^,^^24^ and localized to terminals in the skin.^25^ Interestingly, MOPr mRNA (*Oprm1* gene) is also expressed in keratinocytes.^25^^,^^30^^,^^31^^,^^32^^,^^33^ To examine co-expression of PDGFR-β, PDGF-B and MOPr, with a particular focus on keratinocytes, we used fluorescence *in situ* hybridization (RNAscope, ACDbio). In naive mice, PDGF-B mRNA (*Pdgfb*) and *Oprm1* are co-expressed in keratinocytes, while PDGFR-β mRNA *Pdgfrb* was undetected (Figure 3A). We then examined if five days of repeated morphine i.pl. injections leading to peripheral morphine tolerance could impact PDGF-B expression (Figure 3B). Skin pads from mice injected with saline or morphine i.pl. for five days were collected and processed for RNAscope with *Pdgfb* and *Oprm1* probes. In saline samples, 6.09 ± 1.89% of total keratinocytes co-expressed *Pdgfb* and *Oprm1*(*Pdgfb+Oprm1+*), 5.68 ± 1.73% only expressed *Pdgfb* (*Pdgfb+*)*,* 6.079 ± 2.11% only expressed *Oprm1*(*Oprm1+*), and 82 ± 3.35% did not express either mRNA (*Pdgfb-Oprm1-*, Figure 3C). Interestingly, when comparing samples from the morphine i.pl. group, the number of *Pdgfb+* cells increased to 17.53 ± 2.88%, and *Pdgfb+Oprm1+* cells increased to 27.4 ± 3.09%, while *other* cells decreased to 47.14 ± 3.59% (Figure 3C, N = 3–4/sex/group, two-way ANOVA, Cell type x Treatment, F(3, 39) = 32.87, *p* < 0.0001, Table S3). Thus, the pool of cells expressing *Pdgfb* was increased by i.pl. morphine injections. Furthermore, the amount of *Pdgfb* in *Pdgfb+Oprm1+* keratinocytes was significantly increased in the morphine group compared to the saline group (Figure 3D, Student unpaired 2-tailed *t* test, *p* = 0.0012; Table S3). This finding is also confirmed by an increase in total *Pdgfb* mRNA in skin samples from morphine i.pl. vs. Veh i.pl. mice (Figure 3E, Student unpaired 2-tailed *t* test, *p* = 0.0067; Table S3). These data indicate that repeated i.pl. morphine injections promote an increase in PDGF-B mRNA in keratinocytes that co-express both MOPr and PDGF-B.

### Morphine alters keratinocytes' biophysical properties

**Figure 4:**
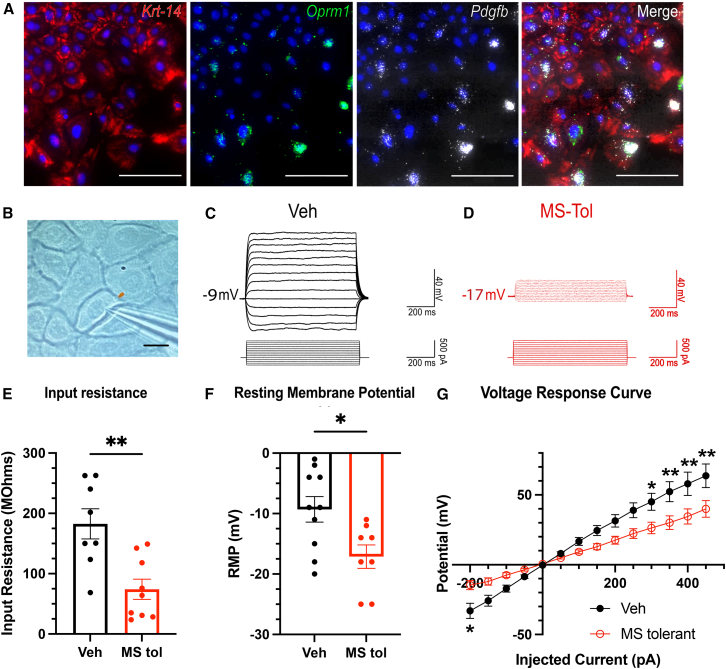
Morphine alters keratinocytes' biophysical properties (A) RNAscope highlights the expression of Krt14, Oprm1 mRNA, and Pdgfb mRNA in primary culture of keratinocytes. (B) Differential interference contrast (DIC) of primary cultured keratinocytes. Scale bar: 25 μm. (C) Representative IV trace obtained from keratinocytes in primary culture obtained from mice treated for five days with saline i.pl. *in vivo*. (D) Representative IV trace obtained from keratinocytes in primary culture obtained from mice treated for five days with morphine i.pl. *in vivo*. (E) Five days of morphine i.pl. decreases keratinocytes input resistance. *N* = 8–9, unpaired Student’s t test. (F) Five days of morphine i.pl. hyperpolarizes keratinocytes as shown by reduced Resting Membrane Potential. *N* = 8–10, unpaired Student’s t test. (G) Comparison of the voltage response curve in primary cultures of keratinocytes obtained from mice treated for five days with saline or morphine i.pl. *in vivo. N* = 9, two-way ANOVA, followed by Sidak’s multiple comparison test. For all figures: ∗*p* < 0.05 ∗∗*p* < 0.01, and ∗∗∗∗*p* < 0.0001 vs. vehicle. Detailed statistics information can be found in Table S4.

To examine whether repeated peripheral morphine administration could directly impact keratinocytes' biophysical properties, we performed patch clamp electrophysiology on primary cultures of keratinocytes (primary KCs) from mice injected with morphine (MS-Tol) or Veh i.pl. for 5 days (Figure 4). Importantly, we confirmed that *Pdgfb* and *Oprm1* mRNA were expressed in our primary KC culture using RNAscope (Figure 4A). Baseline electrophysiological recordings in the Veh group showed an input resistance (IR) of 182.6 ± 25.04MOhms and a resting membrane potential (RMP) of 9.3 mV ±6.70 mV. Keratinocytes are a non-neuronal/non-excitable cell type, explaining these observed depolarized-like values. Additionally, limited prior work using whole cell patch camp electrophysiology in human keratinocytes also reported low polarization, with RMP values ranging from −10 to −30 mV.^58^ It is also possible that our measured RMPs could be influenced by the solution used during the recordings (aCSF). Nevertheless, viability of our recorded cells is supported by the time constant of the membrane of 10.74 ( ±1.55 SEM), indicating that the cells are healthy during recordings (*N* = 7 Veh i.pl., Single exponential fit). Analysis of primary KCs from morphine tolerant mice showed a significantly lower IR relative to their Veh counterparts (Figures 4B–4E, N = 8–9/group, two-tailed unpaired Student’s *t* test *p* = 0.0023; Table S4). Additionally, the average RMP of keratinocytes in the peripheral morphine tolerance condition was significantly more negative (−17.125 mV ±5.46 mV) than that of Veh (9.3 mV ±6.70 mV; Figures 4B–4D and 4F, N = 8–10/group, Student unpaired 2-tailed *t* test, *p* = 0.0169; Table S4). Moreover, voltage responses showed significant differences between the Veh and morphine i.pl. in primary KCs (Figure 4G, *N* = 9/group, two-way ANOVA, injected current x treatment, F (13, 208) = 7.111, *p* < 0.001, Table S4). These data demonstrate that repeated peripheral morphine injections impact the overall biophysical properties of keratinocytes.

### Optogenetic stimulation of keratinocytes mediates peripheral morphine tolerance in a PDGFR-β- and PDGF-B-dependent manner

**Figure 5:**
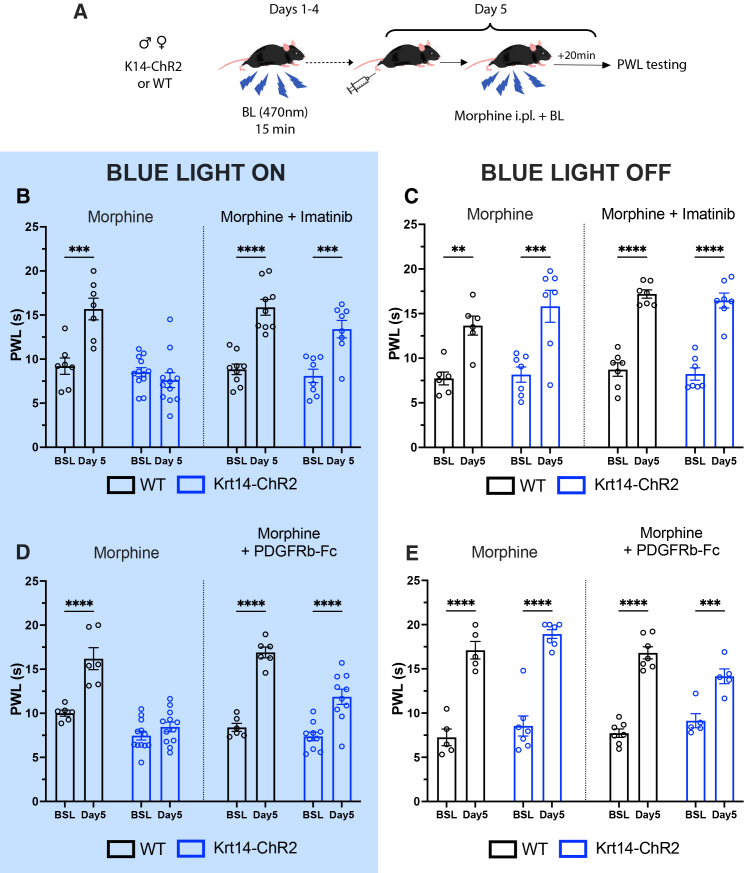
Optogenetic stimulation of keratinocytes mediates peripheral morphine tolerance in a PDGFR-β- and PDGF-B-dependent manner (A) Schematic of the experimental design. See also Figure S4 and Video S1 for supplemental information on the impact of blue light exposure. (B) Blue-light stimulation of keratinocytes for 5 days precludes the antinociceptive effect of a challenge of morphine on day 5, which is restored by a co-administration of morphine + imatinib. N = 7–12 mice/group. (C) Blue-light off controls for Figure 5B. N = 6–7 mice/group. (D) Blue-light stimulation of keratinocytes for 5 days precludes the antinociceptive effect of a challenge of morphine on day 5, which is restored by a co-administration of morphine + the PDGF-B scavenger, PDGFRβ-Fc. N = 6–10 mice/group. (E) Blue-light off controls for Figure 5D. N = 5–7 mice/group. For all Figures: Three-way ANOVA followed by Tukey’s multiple comparisons test. ∗*p* < 0.05, ∗∗*p* < 0.01, ∗∗∗*p* < 0.0001, and ∗∗∗∗*p* < 0.0001. BSL vs. Day 5. WT = Wild-Type. BSL = baseline. Data are expressed as Mean ± SEM. Detailed statistics information can be found in Table S5. See supplemental information in Figure S4.

Evidence of PDGF-B and MOPr co-expression in keratinocytes and the necessity of PDGF-B signaling to mediate peripheral morphine tolerance suggested a mechanistic role for keratinocytes' peripheral opioid tolerance. Earlier work showed that keratinocyte stimulation directly causes action potential firing in several PSN subtypes,^43^ suggesting functional communication between keratinocytes and PSNs, which are known to be involved in tolerance.^40^ We tested whether keratinocyte signaling is sufficient to drive opioid tolerance in the periphery using an optogenetic approach. In transgenic mice, blue-light sensitive channelrhodopsin-2 (ChR2) was genetically expressed in keratinocytes under the control of the Krt14 promoter (Krt14-ChR2 mice).^43^ Krt14-ChR2 mice and wild type (WT) littermates were exposed to blue light for 5 consecutive days for 15 min (BL-ON groups) (Figure 5A). As a control, another cohort of Krt14-ChR2 and WT mice underwent the same procedure, but blue light was kept off (BL-OFF groups). On day 5, mice were injected with i.pl. morphine alone or in combination with imatinib (Figures 5B and 5C), or with PDGFRβ-Fc (Figures 5D and 5E). Strikingly, the morphine analgesic effect was completely abolished in Krt14-ChR2 mice exposed to BL-ON (Figure 5B morphine: N = 7–12/group, 3-way ANOVA, day x genotype x treatment, F (1, 32) = 5.452, *p* = 0.0260, Tukey’s post hoc test BSL vs. Day 5: WT – morphine: *p* < 0.001, K14Chr2 – morphine: *p* = 0.9865, and 5D morphine: N = 6–12/group, 3-way ANOVA, day x genotype x treatment, F (1, 30) = 0.3583, *p* = 0.5539, Tukey’s post hoc test BSL vs. Day 5: WT – morphine: *p* = 0.0008, K14Chr2 – morphine: *p* = 0.9113, Table S5). Importantly, challenge of morphine + imatinib to inhibit PDGFRβ (Figure 5B, morphine + imatinib) or morphine + PDGFRβ-Fc (Figure 5D, morphine + PDGFRβ-Fc) produced a significant antinociceptive effect both in WT littermates and Krt14-ChR2 mice exposed to blue light (BL-ON) for 5 days (Figure 5B, morphine + imatinib: Tukey’s post hoc test BSL vs. Day 5: WT – morphine + Imatinib: *p* < 0.0001, K14Chr2 – morphine + imatinib: *p* = 0.0001; Figure 5D, morphine + PDGFRβ-Fc: Tukey’s post hoc test BSL vs. Day 5: WT – morphine + PDGFRβ-Fc: *p* = 0.0001, K14Chr2 – morphine + PDGFRβ-Fc: *p* < 0.0001; Table S5). However, it is important to note that morphine+PDGFRβ-Fc i.pl. does not fully restore analgesia to levels observed in WT mice (Tukey’s post hoc test, Day5 Krt14-ChR2-MS+Fc vs. WT MS: *p* = 0.0004, and Krt14-ChR2-MS+Fc vs. WT MS + Fc: *p* < 0.0001, Table S5). This suggests that other keratinocyte-derived effectors could be involved in keratinocyte-induced peripheral morphine tolerance. Nevertheless, these data show that: (1) repeated keratinocyte optogenetic activation ablates acute morphine analgesia; and (2) PDGFRβ signaling plays a crucial role in regulating these peripheral keratinocyte effects since morphine analgesia could be restored by the inhibition of PDGFRβ signaling via imatinib or partially restored by scavenging PDGF-B with PDGFRβ-Fc. Control groups that underwent the same procedure but were not exposed to blue light (WT or Krt14-ChR2 BL-OFF) displayed the full analgesic effects of morphine when injected alone or in combination with imatinib (Figure 5C, N = 6–7, 3-way ANOVA, day x genotype x treatment, F (1, 23) = 0.3843, *p* = 0.5414, Tukey’s post hoc test BSL vs. Day 5: WT – morphine: *p* = 0.032, K14Chr2 – morphine: *p* = 0.0072, WT – morphine + Imatinib: *p* = 0.0003, K14Chr2 – morphine + imatinib: *p* < 0.0001, Table S5), or PDGFRβ-Fc (Figure 5E, N = 5–7, 3-way ANOVA, day x genotype x treatment, F (1, 20) = 6.066, *p* = 0.0230, Tukey’s post hoc test BSL vs. Day 5: WT – morphine: *p* < 0.0001, K14Chr2 – morphine: *p* < 0.0001, WT – morphine + PDGFRβ-Fc: *p* < 0.0001, K14Chr2 – morphine + PDGFRβ-Fc: *p* = 0.0015, Table S5). Importantly, recordings of overall behavior during blue light exposure (Video S1), showed that Krt14-ChR2 and WT littermates displayed similar total rearing bouts (Figure S4A, *N* = 6, two-way ANOVA, time x genotype, F (2, 20) = 0.9381, *p* = 0.4079, Table S5). However, we counted significantly more licking (Figure S4B, *N* = 6, two-way ANOVA, time x genotype, F (1.538, 15.38) = 2.400, *p* = 0.1326, Table S5) and forepaw shaking bouts (Figure S4C, *N* = 6, two-way ANOVA, time x genotype, F (1.635, 16.35) = 16.80, *p* = 0.0002, Table S5). Nevertheless, Hematoxylin and Eosin (H&E) staining of skin sections showed no change in overall skin anatomy or epidermal thickness after 5 days of blue light exposure or 5 days of morphine i.pl., suggesting no impact on the structure of the epidermis (Figures S4D and S4E, epidermal thickness; Figure S4E: 1-way ANOVA, F (3, 21) = 1.943, *p* = 0.1537; Table S5). Altogether, these data demonstrate that repeated keratinocyte optogenetic activation is sufficient to induce peripheral morphine tolerance in a PDGF-B/PDGFRβ-dependent manner.

Video S1. Representative behaviors of WT and Krt14-ChR2 mice under blue-light exposure Video recording of the first minutes of blue light exposure in WT (four individuals on the left) and Krt14-Chr2 (four individuals on the right) mice. These videos are related to experiments presented in Figures 5 and S5. Note: light intensity change at 2 min 12 s is due to camera adjustment to light intensity.

### Optogenetic stimulation of keratinocytes does not increase PDGF-B expression

**Figure 6:**
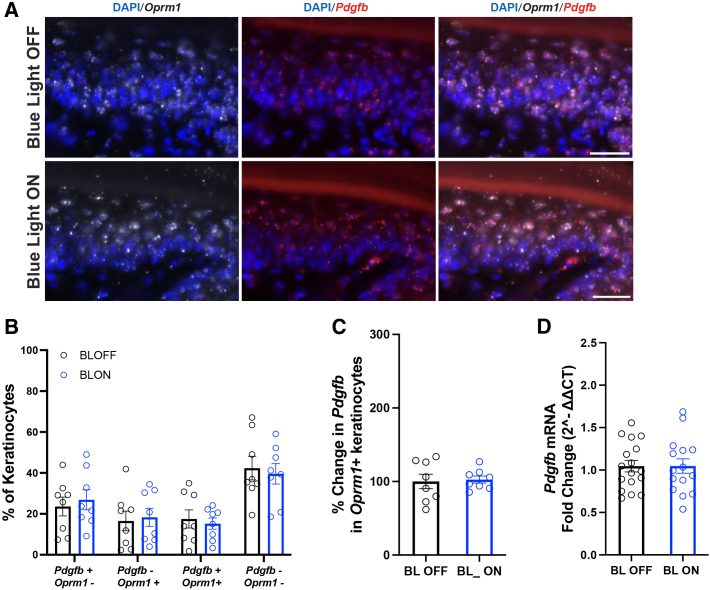
Keratinocyte photostimulation does not alter PDGF-B expression in the skin (A) *In situ* hybridization shows co-expression of *Pdgfb* (red) and *Oprm1* (white) mRNA in epidermal keratinocytes. Scale bars = 20 mm. (B) Quantification of mRNA expression highlights that 5 days of keratinocyte photostimulation in Krt14-ChR2 mice with Blue-Light does not change proportions of keratinocytes expressing *Pdgfb, Oprm1,* or both. “Pdgfb+”: *Pdgfb* expressing cells; “Oprm1+”: *Oprm1* expressing cells; “Pdgfb+Oprm1+”: co-expressing cells; “Pdgfb-Oprm1-”: cells negative for both mRNAs. Two-way ANOVA followed by Uncorrected Fisher’s LSD multiple comparison test. (C) Amount of *Pdgfb* mRNA detected in *Oprm1* cells does not change in Krt14-ChR2 mice exposed to Blue-Light, 15 min per day for 5 days. Unpaired Student’s t test. (D) Total *Pdgfb* mRNA detected in glabrous skin with RT-PCR does not change in Krt14-ChR2 mice exposed to Blue-Light, 15 min per day for 5 days. Unpaired Student’s t test. For all figures. BL ON = Blue Light On, BL OFF = Blue Light Off, *Pdgfb* = PDGF-B mRNA, *Oprm1* = MOPR mRNA. Data are expressed as Mean ± SEM. Detailed statistics information can be found in Table S6.

We examined the impact of optogenetic stimulation of keratinocytes in Krt14-ChR2 mice for 5 consecutive days, on *Pdgfb* expression and distribution in keratinocytes using *in situ* fluorescence hybridization (RNAscope, ACDbio, Figure 6A). Intriguingly, and contrary to what was observed after 5 consecutive days of morphine i.pl. injections (Figure 3), we did not observe a change in the population of cells expressing *Pdgfb, Oprm1,* or both (Figure 6B, *N* = 4/sex/group, two-way ANOVA, Cell type x Treatment, F(3, 42) = 0.1550, *p* = 0.9259, Table S6). The amount of *Pdgfb* in *Pdgfb+Oprm1+* keratinocytes or in bulk extraction of skin mRNA was not changed in the Blue-Light ON group compared to the Blue-Light OFF group (RNAscope: Figure 6C, *N* = 8/group, Student unpaired 2-tailed *t* test, *p* = 0.8109; RT-PCR; Figure 6D, *N* = 8/group, Student unpaired 2-tailed *t* test, *p* = 0.9866; Table S6). These data indicate that 15 min of daily optogenetic stimulation of keratinocytes is not sufficient to promote an increase in PDGF-B mRNA in keratinocytes.

### Inhibition of PDGFRβ restores morphine analgesia and prevents peripheral morphine tolerance in inflammatory and postsurgical pain models

Our study supports a role for PDGFRβ as a target for the prevention of peripheral tolerance from morphine i.pl. injections. Prior work proposed that peripheral and topical opioids are a more effective treatment for lesions that have an inflammatory component.^2^^,^^3^^,^^12^^,^^59^^,^^60^^,^^61^^,^^62^^,^^63^ However, despite growing clinical and pre-clinical evidence that peripherally acting opioids are advantageous,^3^^,^^8^^,^^64^^,^^65^ tolerance has been a significant disadvantage and obstacle to their clinical use at the periphery.^2^^,^^5^^,^^19^^,^^20^ Therefore, to further reinforce the relevance of our findings, we tested whether PDGFRβ inhibition with imatinib could also block tolerance in models of peripheral chronic pain. We chose the model of peripheral inflammatory pain induced by intraplantar injection of Complete Freund’s Adjuvant (CFA) in the hindpaw, and the model of post-surgical pain induced by two consecutive hind paw incisions (double paw incision, DPI).

**Figure 7:**
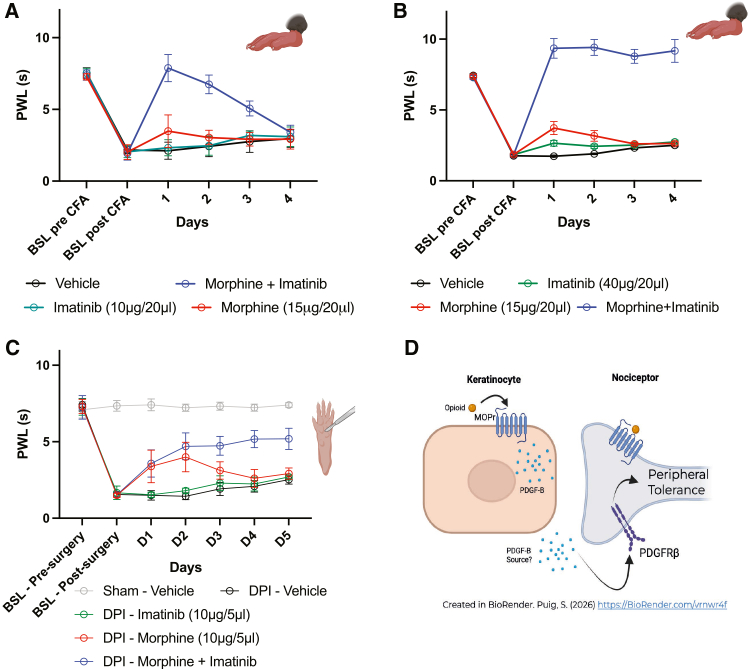
PDGFRβ inhibition restores morphine analgesia and prevents peripheral morphine tolerance in the Complete Freund’s adjuvant (CFA) inflammatory pain model (A) Effect of i.pl. imatinib on peripheral morphine tolerance in the context of chronic peripheral inflammatory pain induced by a CFA hind paw injection. Imatinib at 10 μg (i.pl.) dose does not block peripheral morphine tolerance. (B) Effect of i.pl. imatinib on peripheral morphine tolerance in the context of chronic peripheral inflammatory pain induced by a CFA hind paw injection imatinib at 40 μg (i.pl.) effectively prevents the development of peripheral morphine tolerance. (C) Effect of i.pl. imatinib on peripheral morphine tolerance in the context of chronic peripheral post-surgical pain induced by the double paw incision (DPI) model. Imatinib at 10 μg (i.pl.) dose effectively blocks peripheral morphine tolerance. For all panels: *N* = 3/mice/sex/group. Two-way Repeated Measures ANOVA followed by Tukey’s multiple comparisons test, ∗*p* < 0.05, ∗∗*p* < 0.01, ∗∗∗*p* < 0.0001, and ∗∗∗∗*p* < 0.0001 vs. vehicle (A–B) or DPI-vehicle (C); ##*p* < 0.01, ###*p* < 0.001, and ####*p* < 0.0001 vs. DPI-vehicle (C); Data are expressed as mean ± s.e.m. Detailed statistics information can be found in Table S7. See supplemental information in Figure S5. (D) Model illustrating identified mechanisms of peripheral morphine tolerance. We propose that repeated peripheral morphine administration causes the release of PDGF-ligand from peripheral sources, such as keratinocytes or immune cells, which in turn activates PDGFRβ in PSN nociceptors and activates signaling pathways that cause peripheral tolerance.

We first conducted dose-response studies of morphine i.pl. analgesia to establish the morphine i.pl. efficacious dose (Figure S5). Importantly, in the CFA model, the volume injected in inflamed paws was increased to 20 μL to ensure proper distribution of injected drugs (Figure S5A). In CFA mice, we found that combining morphine with imatinib dose-dependently restored morphine i.pl. analgesia (Figure S5A ipsilateral: *N* = 3/sex/group, two-way ANOVA, treatment x morphine dose: F (6.506, 43.37) = 90.15. *p* < 0.0001; Figure S5A contralateral: *N* = 3/sex/group, two-way ANOVA, treatment x morphine dose: F (8.326, 55.51) = 6.537. *p* < 0.0001, Table S7). In DPI, increasing the dose of morphine was sufficient to restore peripheral morphine analgesia (Figure S5B ipsilateral: *N* = 5/group, two-way ANOVA, treatment x morphine dose: F (2.167, 17.34) = 2.704. *p* < 0.0001 for the morphine dose; Figure 7B contralateral: *N* = 5/group, two-way ANOVA, treatment x morphine dose: F (3.057, 24.45) = 0.1754. *p* < 0.0001 for the morphine dose, Table S7). In both models, doses were chosen for their ability to induce analgesia and bring thermal thresholds to levels of the contralateral paws. Note that in the DPI model, the contralateral paw, which had surgery a week prior to injections, had thresholds lower than those of Sham mice, which is expected in this mode (Figures 7 and S5B).^66^ In CFA treated mice, morphine was used at 15μg/injection whereas in DPI mice, morphine was used at 10μg/injection. We then tested the ability of imatinib to prevent peripheral morphine tolerance in both models. Interestingly, in the CFA model, while 10 μg imatinib did not affect peripheral morphine tolerance (Figure 7A: *N* = 3/sex/group, two-way ANOVA, treatment x time: F (10.01, 66.71) = 61.68, *p* < 0.0001, Table S7), 40 μg completely blocked it as morphine i.pl. analgesia was fully maintained during the four days of morphine+imatinib co-administration (Figure 7B: *N* = 3/sex/group, two-way ANOVA, treatment x time: F (6.505, 69.38) = 56.08. *p* < 0.0001, Table S7). Of note, 40 μg imatinib in 20 μL of Veh brings imatinib to the same concentration of imatinib that effectively blocked tolerance in naive animals (e.g., 10μg/5 μL, Figure 1), suggesting that this concentration is required to block peripheral tolerance. In the DPI model, results show the dose of 10μg/5 μL of imatinib, similar to the dose used in naive animals (Figure 1), blocked the development of peripheral morphine tolerance (Figure 7C: *N* = 3/sex/group, two-way ANOVA, treatment x time: F (15.12, 94.49) = 37.90. *p* < 0.0001, Table S7). These data show that in models of chronic peripheral inflammatory and post-surgical pain, (1) the peripheral analgesic potency of morphine is reduced and (2) imatinib can effectively restore morphine analgesia and prevent peripheral morphine tolerance.

## Discussion

We show that PDGFRβ inhibition using imatinib or a PDGFRβ mAb completely blocks peripheral tolerance to morphine. In naive mice, repeated, but not acute, peripheral administration of PDGF-B ligand induces peripheral morphine tolerance, and repeated, but not acute, i.pl. morphine injections phosphorylate PDGFRβ in PSNs. These data strongly support that PDGF-B/PDGFRβ signaling is both necessary and sufficient to cause peripheral opioid tolerance. To highlight the potential for PDGFRβ to be a promising target, we also show that imatinib, an FDA-approved PDGFRβ inhibitor, promotes peripheral analgesia and blocks peripheral tolerance in inflammatory and post-surgical pain conditions. Imatinib is used in humans for the treatment of malignancies at daily doses that are up to 400 times higher than used in our study and is well tolerated,^67^^,^^68^^,^^69^^,^^70^ suggesting that it will be similarly well tolerated for the treatment of pain in humans.

Overall, we are the first to provide a mechanistic explanation for peripheral tolerance. Underlying mechanisms uncovered here include dynamic interactions between keratinocytes, peripheral PDGF-B release, and PDGFRβ signaling. We show that (1) keratinocytes express PDGF-B and MOPr (but not PDGFRβ) and that repeated peripheral morphine administration increases PDGF-B expression in MOPr-expressing keratinocytes, (2) peripheral morphine administration causes profound changes in the biophysical properties of keratinocytes, and (3) selective keratinocyte photostimulation is sufficient to cause peripheral morphine tolerance in a PDGF-B/PDGFRβ-dependent manner. While direct evidence of PDGF-B release remains to be established a role for keratinocytes in peripheral opioid signaling and peripheral tolerance, this is the first study to identify druggable targets that could facilitate the transition from systemic to peripheral opioid delivery in cases of peripheral injury.

Our results challenge the notion that peripheral and central tolerance are fundamentally distinct.^13^^,^^71^^,^^72^ This distinction was previously suggested by studies using locally applied opioids in animals already tolerant to systemic opioids,^72^^,^^73^ but conflicting findings have left the mechanisms of peripheral tolerance unresolved.^18^^,^^74^ Moreover, the role for peripheral PSN MOPrs in tolerance and analgesia remains controversial; prior assumptions suggested that peripheral opioids primarily acted on central MOPrs,^75^ as opioids induce analgesia when directly injected into the CNS in rodents^76^^,^^77^ and humans.^78^ However, it is now accepted that opioids cause analgesia peripherally via action on MOPrs in PSNs,^79^^,^^80^ decreasing their excitability in peripheral tissues.^3^^,^^6^^,^^7^^,^^81^^,^^82^^,^^83^^,^^84^ Our data demonstrate that i.pl. morphine induces localized ipsilateral analgesia without contralateral effects, supporting peripheral MOPr action without central penetration. Additionally, reports that peripherally administered opioids have limited CNS bioavailability reinforce the idea that opioids act locally when delivered.^12^^,^^85^

Nevertheless, the role of peripheral MOPrs in tolerance and analgesia remains unresolved. Studies where MOPr was conditionally knocked out from PSNs (using Pirt-cre^86^ and Advillin-cre^87^ mice crossed with Oprm1 floxed mice), reported the absence of analgesia following systemic opioid injections. In contrast, other studies using alternative cre driver lines (TrpV1-cre^40^ and NaV1.8-cre^88^) found that analgesia was preserved, and tolerance was either blocked or not altered, demonstrating the complexity of this issue.

We are the first to examine peripheral tolerance mechanisms using local morphine injections, enabling direct assessment of ipsilateral analgesia without confounding contralateral or central effects seen with systemic opioids.^40^^,^^41^^,^^87^^,^^88^ Importantly, we show that peripherally, MOPr is also expressed in non-neuronal cells such as keratinocytes,^25^^,^^30^^,^^31^^,^^32^^,^^33^ and propose that MOPr signaling in these cells may be critical for peripheral tolerance, an element that may resolve previous controversies. Nonetheless, we acknowledge that the necessity of keratinocyte MOPr signaling in peripheral tolerance requires further investigation and is the focus of ongoing studies.

A major finding of this study is that co-administration of morphine with imatinib completely blocks peripheral tolerance and does so in models of inflammatory and post-surgical pain. Since imatinib targets other RTKs in addition to PDGFRβ,^53^ we confirmed that this effect was specific to PDGFRβ using a selective PDGFRβ mAb, whose co-administration with morphine also blocked peripheral morphine tolerance. Blockade was also observed with a PDGFRβ-Fc chimera protein, further confirming the role of PDGF-B in tolerance.^89^ Thus, we propose that imatinib effects on peripheral tolerance are mediated via its actions on PDGFRβ.

We previously reported^26^^,^^29^ that PDGFRβ is expressed in the soma and cutaneous nerve endings of peripheral sensory neurons, where it is enriched in small diameter and non-myelinated fibers,^26^ where MOPrs are also expressed.^23^^,^^90^^,^^91^ Thus, combining our finding that repeated (but not acute) intraplantar morphine injections induce PDGFRβ phosphorylation in PSNs, and repeated (but not acute) recombinant PDGF-B intraplantar injections cause a peripheral tolerance-like state, we propose that repeated peripheral PDGFRβ stimulation in PSNs mediates peripheral tolerance. We also speculate that the activation of PDGFRβ signaling in PSNs may alter MOPr signaling in these neurons, possibly precluding signaling mechanisms necessary for peripheral analgesia, and that imatinib or PDGFRβ mAb prevents tolerance by inhibiting PDGFRβ signaling in PSNs. PDGFRβ modulation of MOPr signaling in PSNs is supported by the fact that GPCRs and RTKs are known to crosstalk^42^^,^^92^^,^^93^^,^^94^^,^^95^^,^^96^ and that RTK activation upon MOPr signaling has been demonstrated *in vitro.*^22^^,^^94^^,^^97^

Our data show that PDGF-B/PDGFRβ signaling is both necessary and sufficient for peripheral morphine tolerance, suggesting peripheral and central tolerance share common mechanisms.^22^^,^^27^ Prior work showed that intrathecal morphine tolerance also involves PDGFRβ and PDGF-B release. Together with our work, this shows that PDGF-B release in the spinal or skin nerve ending environment of PSNs is sufficient to cause tolerance. Thus, we propose a model in which morphine, delivered intrathecally or cutaneously, induces PDGF-B release, activating PDGFRβ signaling in PSNs and leading to tolerance (central or peripheral, respectively).

The fact that PDGFRβ mAb co-administration with morphine i.pl. prolonged the duration of peripheral analgesia, but not the extracellular ligand scavenger, PDGFRβ-Fc, suggests that PDGFRβ signaling influences the duration of morphine analgesia through mechanisms independent from PDGF-ligand release. Conversely, PDGFRβ-Fc completely blocks peripheral morphine tolerance but does not alter acute morphine analgesia. This suggests that PDGFRβ-induced tolerance involves PDGF-ligand-dependent mechanisms. The necessity of PDGFRβ in PSNs for tolerance, and whether recruited PDGFRβ downstream pathways differ depending on the mode of MOPr-PDGFRβ transactivation (direct or ligand-dependent), is the focus of ongoing studies.

Another important finding of our study is that selective *in vivo* photostimulation of keratinocytes using optogenetics in Krt14-ChR2 mice is sufficient to induce peripheral morphine tolerance, even in the absence of repeated opioid injections, and this was precluded by acute co-administration of morphine+imatinib and partially precluded by co-administration of morphine + PDGFRβ-Fc. This indicates a previously unrecognized major role of keratinocytes/PDGFRβ signaling in peripheral tolerance. Importantly, the partial preclusion of keratinocyte-mediated peripheral tolerance with the PDGFRβ-Fc suggests that other keratinocyte-released factors are involved in this response and is consistent with prior work.^43^^,^^45^^,^^48^^,^^50^ Nevertheless, these findings support that keratinocytes are important components of the peripheral circuitry that underlies opioid tolerance. This is also shown by profound alterations in keratinocytes' biophysical properties caused by i.pl. morphine as measured by patch-clamp electrophysiology. While the mechanism underpinning these changes remains unclear, the hyperpolarized state may stem from an increase in potassium conductance, a phenomenon that may account for the decrease in membrane resistance. However, G protein-coupled inwardly rectifying potassium channels (e.g., Kir2.1) activated by MOPr in neurons are unlikely to account for the shift in resting potential in keratinocytes as the KCNJ2 transcript coding for this channel was undetected in keratinocytes in prior studies.^37^ Future work will examine mechanisms that may drive morphine mediated changes in keratinocytes biophysical properties and whether they could be involved in the etiology of peripheral tolerance.

Two distinct interventions (morphine i.pl. injections and keratinocyte photostimulation) were shown to be sufficient to induce PDGF-B/PDGFRβ-dependent tolerance. This finding is interesting as ChR2 generally causes membrane depolarization and action potential firing in neurons, whereas morphine activates MOPr, which is coupled to an inhibitory Gi/o signaling in neurons (e.g., inhibiting cAMP production and action potentials). However, neuronal responses to opsins and MOPr may differ in non-neuronal cells. Thus, combined with our data showing that peripheral tolerance is selectively caused by PDGF-B/PDGFRβ signaling, these findings suggest that peripheral tolerance is independent of MOPr’s inhibitory properties and may involve specific signaling mechanisms that overlap with the actions of opsins in keratinocytes, such as, for example, intracellular calcium signaling. This concept is further supported by the observed increased polarization and leakier epithelium in keratinocytes from peripherally tolerant mice, consistent with repeated keratinocyte photostimulation being sufficient to cause peripheral morphine tolerance. Thus, keratinocyte hyperactivity/phenotypic shift may be a causal upstream event in peripheral tolerance, e.g., through the release of factors that cause peripheral tolerance.

Consistent with this idea and with prior published work,^36^^,^^37^^,^^38^^,^^98^ we show that keratinocytes express PDGF-B but not PDGFRβ, and that peripheral morphine administration for five days increases PDGF-B expression in keratinocytes that co-express MOPr and PDGF-B. Thus, together with the blockade of tolerance by PDGF-B inhibition, our findings strongly suggest the necessity of PDGF-B release for peripheral tolerance. Because keratinocytes are known to release factors that change nociceptor signaling,^43^^,^^45^^,^^48^^,^^50^^,^^99^^,^^100^ prior work combined with our current findings makes us now speculate that keratinocyte stimulation (by morphine or blue light) promotes the release of PDGF-B, which drives opioid tolerance. However, an important consideration is that daily photostimulation of keratinocytes did not increase PDGF-B transcription, despite blocking keratinocyte-mediated peripheral tolerance with the PDGF-B scavenger, suggesting repeated keratinocyte photostimulation is insufficient to drive gene expression patterns evoked by repeated morphine injections. Nevertheless, because ligand release still seems to be induced, there remains the possibility that PDGFB protein levels are regulated post-transcriptionally. Moreover, growth factor secretion can involve vesicular or extracellular matrix release mechanisms.^101^^,^^102^ These different possibilities and the mechanisms by which MOPr or ChR2 stimulation may cause release of PDGF-B remain to be elucidated.^101^^,^^102^

Importantly, there also remains the possibility that PDGF-ligand may be released by other cells recruited by keratinocyte signaling upon peripheral MOPr stimulation. Notably, opioids can reshape and promote the recruitment of macrophages and T cells,^103^^,^^104^ and these activated immune cells are known to express PDGF-ligands.^105^ Possible involvement of immune cells in peripheral opioid analgesia and tolerance is supported by our observation that levels of analgesia were altered in the context of inflammatory and post-surgical pain. Interestingly, i.pl. morphine+imatinib restored full peripheral morphine analgesic efficacy in CFA-inflamed hindpaws. This suggests that the observed alteration of peripheral analgesic efficacy in inflammatory conditions involves PDGFRβ signaling mechanisms.

Our proposed model (Figure 7D) is one where peripheral opioids engage PDGF-B/PDGFRβ signaling to cause peripheral tolerance by (1) releasing PDGF-B, and (2) activating PDGFRβ on peripheral nociceptors to mediate peripheral tolerance. The potential consequences of morphine action on MOPr in keratinocytes, the source(s) of PDGF-B, and the mechanisms of PDGFRβ signaling-mediated peripheral tolerance are under investigation. Nevertheless, we are the first to shed light on tangible clues for the mechanisms of peripheral opioid tolerance and provide druggable targets that could help shift opioid delivery from systemic to peripheral in the context of peripheral injury.

In summary, we demonstrate that PDGF-B/PDGFRβ signaling is an essential signaling mechanism involved in peripheral morphine tolerance. We found that keratinocyte stimulation is sufficient to induce peripheral opioid tolerance, highlighting that keratinocytes may be an integrative component of the peripheral somatosensory circuit that mediates opioid signaling and peripheral tolerance. Our work opens avenues of research on the potential of targeting keratinocytes and PDGFRβ to preserve peripheral morphine efficacy over time and enable the use of peripheral opioids for the treatment of local peripheral pain. If successful, this approach could be leveraged to shift opioid administration from central/systemic to localized/topical, thus circumventing the dangers of opioid addiction and abuse.

### Limitations of the study

Our pharmacological manipulations do not provide direct evidence that morphine acts specifically on keratinocytes via MOPr, or that keratinocytes are the definitive source of PDGF-B in this context. Future studies employing conditional genetic ablation of MOPr or PDGF-B in keratinocytes are needed to clarify these mechanistic pathways. Mechanisms of MOPr-mediated PDGF-B release will also be addressed upon the confirmation of PDGF-B release directly by MOPr-expressing cells. Another limitation is that the optogenetic activation of keratinocytes may not depolarize these cells in the same manner as neurons. Additionally, we find that keratinocyte photostimulation seems to mimic the effect of repeated morphine injections, which, in neurons, acts on the Gi/o MOPr to inhibit membrane depolarization. Therefore, the relevance of these parallels should be interpreted with caution. Finally, although we establish a critical role for PDGFRβ in peripheral tolerance and confirm its expression and phosphorylation by i.pl. morphine in PSNs, our study does not demonstrate the necessity of PDGFRβ specifically in PSNs for this phenotype, nor does it elucidate the precise molecular mechanisms by which PDGFRβ interferes with MOPr signaling in these neurons. Addressing these gaps in future studies will be crucial for fully understanding the cellular and molecular architecture of peripheral opioid tolerance.

## Resource availability

### Lead contact

• Requests for further information and resources should be directed to and will be fulfilled by the lead contact, Stephanie Puig (stephanie.puig1@umassmed.edu).

### Materials availability

• This study did not generate new unique reagents.

### Data and code availability

#### Data

• All data reported in this paper will be shared by the lead contact upon request.

#### Code

• This paper does not report original code.

#### Additional information

• Any additional information required to reanalyze the data reported in this paper is available from the lead contact upon request.

## Acknowledgments

We thank Ms. Bahhiyah Jefferson, research technician in the Albers laboratory at the 10.13039/100007921University of Pittsburgh for her technical assistance with experiments for this project. We also thank Ms. Kennedy O’Hara, first-year graduate student in the Morningside Graduate School at UMass Chan, for her technical assistance with experiments for this project.

For this work, S.P. was supported by the National Institute of Health (10.13039/100000002NIH) R21DA051636. K.M.A. was supported by 10.13039/100000002NIH R21DA051636, R01AR0777341, and R01DK124955. R.W.L. was supported by 10.13039/100000002NIH R01DA051390 and R01DA06124. Z.F. was supported by The 10.13039/100000945Pittsburgh Foundation (J.F. and N.A. Emmerling Fund of the Pittsburgh Foundation, FPG00043), the 10.13039/100023044Commonwealth of Pennsylvania (PA-HEALTH to Z.F.), and 10.13039/100000002NIH R21AA028800; R01ES034037, and R01DA061243.

## Author contributions

S.P.: conceptualized and designed the study; S.P.: obtained funding with help from K.M.A.; L.P., S.P., S.A.M., A.K.M., M.F., A.K., T.L., and A.B.: performed experiments; S.P. and L.P. analyzed data; M.G., K.M.A., Z.F., R.W.L., G.M., and S.F.R.: helped with analysis; L.P. and S.P. wrote the manuscript.

## Declaration of interests

Z.F. is funded by an investigator-initiated award from UPMC Enterprises. All other authors declare no competing interest.

## Declaration of generative AI and AI-assisted technologies in the writing process

During the preparation of this work, the author(s) used the UMass Chan AI tool (OpenAI GPT-4.1-mini) to correct English grammar in some paragraphs. After using this tool or service, the author(s) reviewed and edited the content as needed and take full responsibility for the content of the publication.

## STAR★Methods

### Key resources table

**Table:** 

REAGENT or RESOURCE	SOURCE	IDENTIFIER
**Antibodies**		

PDGFRβ monoclonal neutralizing antibody	Invitrogen	Cat #16-1402-82; RRID: AB_469070
IgG2a kappa Isotype Control antibody	Invitrogen	Cat #16-4321-82; RRID: AB_410156
HRP conjugated anti β-actin mAb	Sigma	Cat #A3854; RRID: AB_262011
PDGFRβ rabbit polyclonal antibody	ThermoFisher	Cat #MA5-15143; RRID: AB_10985851
Y1021-PDGFRβ rabbit polyclonal antibody	Bioss	Cat #bs-3322R; RRID: AB_10856220
HRP conjugated goat anti-rabbit antibody	Cell Signaling	Cat. #7074S; RRID: AB_2099233

**Chemicals, peptides, and recombinant proteins**		

Morphine sulfate	Sigma	Cat #6211-15-0
Imatinib	LC Laboratories	Cat #I-5508
Recombinant PDGF-BB peptide	R&D Systems	Cat. #520-BB
PDGFRβ-Fc scavenger	R&D Systems	Cat. #1042-PR
10% β-cyclodextrin sulfobutyl ether (Captisol)	Sigma Aldrich	Cat. #A11765-25G
1X PBS	Sigma-Aldrich	Cat. #P5493
Trizol	Invitrogen	Cat. #15596026
Chloroform	Sigma Aldrich	Cat. #319988
TaqMan Gene expression MasterMix	ThermoFisher	Cat. #4369016
Paraformaldehyde 4%	Fisher Scientific	Cat. #J19943K2
DAPI	ACD Bio	Cat. #323108
HBSS	Fisher Scientific	Cat. # 14170112
Ethanol 100%	Fisher Scientific	Cat. #01-335-389
Sucrose	Sigma	Cat. # S8501
Penicillin-Streptomycin 5,000U/ml	Fisher Scientific	Cat. #15070063
Amphotericin B	Sigma Aldrich	Cat. #A2942
Trypsin-EDTA	Fisher Scientific	Cat. #25300054
Defined Keratinocyte Serum Free Basal Medium	Fisher Scientific	Cat. #10744019
Defined Keratinocyte SFM (1X)	Fisher Scientific	Cat. #10744019
Epidermal growth factor	Fisher Scientific	Cat. #53003-018
Collagen	Fisher Scientific	Cat. #NC1558174
NaCl	Sigma Aldrich	Cat#S7653
KCl	Sigma Aldrich	Cat#P9541
NaH2PO4⋅H2O	Sigma Aldrich	Cat. # S9638
MgCl2	Sigma Aldrich	Cat. #M9272
CaCl2	Sigma Aldrich	Cat. #C7902
NaHCO3	Sigma Aldrich	Cat. #S6014
D-Glucose	Sigma Aldrich	Cat. # AMBH2D6F3F7C
K-methanesulfonate	Sigma Aldrich	Cat. #83000
Complete Freund Adjuvant	Sigma-Aldrich	Cat.#F5881

**Critical commercial assays**		

Directzol RNA isolation kits	Zymo Research	Cat. #R2050
RNA to cDNA kit	ThermoFisher	Cat. #438746
Pdgfb probe (PCR)	ThermoFisher	Mm00440677_m1
β-actin-FAM (PCR)	ThermoFisher	Mm02619580_g1
RNAscope kit V2	ACDBio	Cat. #323100
Pdgfb probe, Mm-PDGF-B-C1 (RNAscope)	ACDBio	Cat. #42451
Pdgfrβ probe, Mm-PDGFr-B-C2 (RNAscope)	ACDBio	Cat. #411381-C2
Oprm1, Mm-Oprm1-C3 (RNAscope)	ACDBio	Cat. #315841-C3
Dispase II	Sigma Aldrich	Cat. #4942078001
RIPA IP Lysis buffer	ThermoFisher	Cat. #87787
Protease Inhibitor cocktail	Sigma	Cat. #13786
Phosphatase inhibitor cocktail 2	Sigma	Cat. #P5726
Phosphatase inhibitor cocktail 3	Sigma	Cat. #P0044
BCA Assay	ThermoFisher	Cat. #A55864
4X Laemmli Sample Buffer	Biorad	Cat. #1610747
6%, Tris-Glycine	ThermoFisher	Cat. #XP00065

**Experimental models: Organisms/strains**		

C57BL/6J mice	Jackson laboratory	# 000664
Krt14-Cre mice	Jackson laboratory	# 004782
Ai32 mice	Jackson laboratory	# 012569

**Software and algorithms**		

PatchMaster 2.15	HEKA Elektronik	N/A
FitMaster 2.15	HEKA Elektronik	N/A
Prism 9.5	GraphPad	https://www.graphpad.com/
RStudio (version 2023.06.0-421)	RStudio Team	https://posit.co/products/open-source/rstudio/
R (version 4.3.1)	R Core Team (2023)	https://www.R-project.org/.
Image J Fiji	Image J, NIH, USA	http://imagej.org
QuPath 0.6.0-arm64	QuPath	

### Experimental model and study participant details

#### Animals

All experiments were performed on mice 8-14 weeks old. All procedures were approved and performed in compliance with the Institutional Animal Care and Use Committee at Boston University and University of Massachusetts Chan Medical School (IACUC #IPROTO202300000062).

Mice were originally purchased from the Jackson laboratory (C57BL/6J (stock # 000664), Krt14-Cre (stock# 004782), Ai32 (stock #012569)). Animals were grouped and housed in a 12/12 light cycle (7 am lights on, 7 pm lights off). Water and rodent chow were provided *ad libitum* throughout the experiment. Animals were habituated to the laboratory environment for 1 week prior to experimental testing or breeding. For optogenetic experiments, we generated Krt14-ChR2 mice by crossing mice that express Cre-recombinase under the control of the Krt14 promoter (Krt14-Cre) with mice that express the blue-light-sensitive channelrhodopsin-2 (ChR2) under the control of the CAG promoter inserted into the Rosa26 locus (Ai32) producing Krt14-ChR2 mice that express ChR2 in Krt14+ keratinocytes(40). F2 generation mice were used to obtain Cre allele heterozygous and either ChR2 heterozygous or homozygous mice. Littermate animals not expressing the ChR2 allele were used as wild-type (WT) controls.

Our study included both male and female mice and was powered to detect behavioral sex differences on peripheral tolerance behaviors. No specific sex effects were detected in initial experiments, therefore, analysis of following behavioral characterizations were done with both sexes merged.

### Method details

#### Drugs

Morphine sulfate was obtained from Sigma (cat. number: 6211-15-0), Imatinib from LC Laboratories (Cat. # I-5508), recombinant rat PDGF-BB peptide (Cat #520-BB), recombinant mouse PDGFRβ-Fc scavenger (Cat. # 1042-PR) from R&D Systems, PDGFRβ monoclonal antibody (PDGFRβ mAb) from Invitrogen (Cat #16-1402-82), IgG2a kappa Isotype Control antibody from Invitrogen (Cat #16-4321-82). Drugs were either dissolved in a solution of filtered (0.22 μm) 10% β-cyclodextrin sulfobutyl ether (Captisol, CyDex) in 0.9% saline (for Imatinib, PDGFRβ-Fc, and PDGFRβ mAb), or in filtered 4mM HCl in 0.9% saline (for PDGF-BB peptide).

#### Intraplantar (i.pl.) drug administration

All drugs were injected subcutaneously in the same region of plantar skin of one hind paw of awake animals that were lightly restrained. The volume of injection was 5μl in naïve mice, or 20μl in CFA mice, using a 30-gauge needle and 20μl Hamilton syringe. The paw side was randomized within and across experiments. Doses per injection were as follows: Morphine Sulfate: 5-20μg, Imatinib: 10-40μg, PDGF-B peptide: 0.25μg, PDGFRβ-Fc: 250ng, PDGFRβ: mAb 0.1μg, IgG2: 0.1μg. Doses used were chosen after performing dose-responses – see Figures 7 and S1.

#### Nociceptive testing

##### Thermal nociception assay

Mice were habituated to a Hargreaves apparatus (IITC Life Sciences Inc., Woodland Hills, CA) on a tempered glass maintained at 30°C for 90 minutes per day for 2 days and prior to behavioral testing. Intensity of the targeted light source was adjusted to generate baseline responses at ∼8 seconds on average. A cut-off of 20 seconds was set to avoid tissue damage. Thermal nociceptive thresholds were recorded following habituation by measuring paw withdrawal latencies (PWL) to a light source focused on the plantar surface of each hind paw. Recorded behaviors for latency included withdrawal, licking, biting, or shaking of the targeted hind paw. Measurements were repeated three times per hind paw, with at least 1-minute intervals and averaged for each hind paw.

PWLs were measured every day before and 20 minutes after i.pl. injections, to follow the development of thermal peripheral tolerance and thermal opioid induced hyperalgesia (OIH). Development of tolerance is characterized by a reduction of PWLs post morphine i.pl. injection overtime. Development of OIH is characterized by a reduction of baseline PWLs over time. OIH is calculated by subtracting initial baseline (BSL) PWL to the baseline measured on day five (morphine not onboard, mice have received four doses of morphine i.pl. for the prior four days).

##### Mechanical nociception assay

Mice were habituated to opaque experimental plexiglass compartments placed on an elevated grid for 90 minutes per day for 2 days and prior to behavioral testing. On test days, mechanical nociceptive thresholds were recorded following habituation by measuring paw withdrawal thresholds (PWT) using vonFrey filaments applied on plantar surface of each hind paw. We use the simplified up-down method (SUDO^106^) and filament number 6 was used as starting filament. Recorded behaviors for nociceptive response included withdrawal, licking, biting, or shaking of the tested hind paw. Measurements were spaced by at least 1-minute intervals and averaged for each hind paw.

PWTs were measured every day before and 20 minutes after i.pl. injections, to follow the development of mechanical peripheral tolerance and mechanical OIH. Development of tolerance is characterized by a reduction of PWTs post morphine i.pl. injection overtime. Development of OIH is characterized by a reduction of baseline PWLs over time. OIH is calculated by subtracting initial baseline (BSL) PWT to the baseline PWT measured on day five (morphine not onboard, mice have received four doses of morphine i.pl. for the prior four days).

#### Peripheral chronic pain models

##### Peripheral chronic inflammatory pain

Peripheral chronic inflammatory pain was induced using Complete Freund’s Adjuvant (CFA) (Sigma, Cat. #F5881). Mice were briefly anesthetized with 3% isoflurane via a nose cone, the paw was disinfected with 70% ethanol and betadine (McKesson, Cat. #1073830), 3 times and 20μL of 100% CFA was injected intraplantarly into the left hind paw using an insulin syringe with a 28-gauge needle. Control animals received an equivalent volume of sterile saline. Following injection, mice were returned to their home cages and monitored daily.

##### Peripheral post-surgical pain

Post-surgical pain was induced using the double paw incision model. Mice were anesthetized with 3% isoflurane delivered via a nose cone and placed on a heating pad to maintain body temperature. The right hindpaw was disinfected with 70% ethanol and betadine 3 times. A ∼5 mm longitudinal incision was made through the skin and fascia of the plantar surface using a sterile scalpel blade (No. 11) (Fischer Scientific, Cat. # 12460452). The incision started approximately 2–3 mm from the proximal edge of the heel and extended toward the toes, avoiding major footpads. The underlying plantar flexor muscle was gently elevated and incised longitudinally using a sterile scalpel blade (No. 11). The skin was then closed with two double sutures using PolySyn Undyed Braided Suture 4-0 (Medline, Cat. #SUS386B). Following surgery, mice were allowed to recover in a warmed cage and then returned to their home cages once fully awake. Sutures were removed 3 days after surgery. Surgery of the first hindpaw only causes hyperalgesia for 3-5 days,^107^ which would not allow to test development of peripheral tolerance. A second surgery on the opposite hindpaw was therefore necessary, to prolong hyperalgesia for up to 21 days.^66^^,^^108^ Therefore, seven days after the first surgery, the same procedure was repeated to the left hindpaw. Sham animals only went through anesthesia, without any incision. All i.pl. injections and tests of impact of imatinib on peripheral tolerance in this post-surgical pain model were conducted in the paw that underwent the second surgery.

#### Experimental workflow

Development of peripheral tolerance with morphine: Mice were randomly assigned to drug groups. Development of tolerance and testing of pharmacological agents were performed in a 2x2 design by injecting either vehicle, morphine alone, the pharmacological agent alone, or morphine combined with the pharmacological agent. Mice were habituated to the Hargreaves apparatus or vonFrey elevated grid for 60 to 90 min before drug injections. Baselines were acquired on day 0. On days 1-5, animals were injected intraplantarly (i.pl.) with an assigned drug, placed on the Hargreaves apparatus and PWL measured 20 minutes later. The 20-minute time point of testing was determined through a time course experiment to ascertain the peak analgesic effects of morphine administered i.pl. On day 5, animals were euthanized and tissue collected according to tissue collection procedures.

Development of peripheral tolerance with blue light: On day 0, Krt14-ChR2 mice and WT littermates were habituated to the Hargreaves apparatus for 60 to 90 min before light exposure and drug injections. Following habituation, baseline PWL was measured using our Hargreaves apparatus. On days 1-4, animals were exposed to a blue light using a LED covered tray (170 Lumens, 475 nm wavelength) placed underneath the tempered glass surface. Blue light exposure lasted 15 minutes per day. On day 5, animals were injected in one hind paw (ipsilateral, ipsi) with either morphine or morphine combined with the tested agent, before being exposed to 15 min blue light exposure and paw withdrawal latency testing 5 minutes later. Control groups underwent the same procedure with the blue light off.

#### Tissue collection

Mice were anesthetized with 4% isoflurane vapor until respiration ceased followed by intracardiac perfusion. The abdomen was incised to expose the heart, a butterfly needle inserted into the left ventricle and the right atrium was cut to create an open circuit. Filtered 1X phosphate-buffered saline (PBS) was pumped through the circulatory system to remove all blood from the body. Hind paw skin was removed and cut in half longitudinally. One half was placed in 1mL of Trizol for RNA isolation and the other half was placed in 2% paraformaldehyde (PFA) at 4°C for 48 hours for fluorescence *in situ* hybridization analysis.

#### Reverse transcription - Polymerase chain reaction

Skin samples were homogenized in Trizol (Invitrogen, Cat. #15596026), incubated in 0.2mL chloroform for 2 min and centrifuged (15 min, 12,000 xg et 4°C) for phase separation. Directzol RNA isolation kits (Zymo Research, Direct-zol, cat #R2050) were used to isolate genomic DNA-free RNA. cDNA conversion was performed using the high-capacity RNA to cDNA kit (ThermoFisher, cat #438746). Input amount of RNA used was 2ug. cDNA reactions were done using a Thermocycler programmed at 37°C for 1 hour, 95C for 5min and held at 4°C. For RT-PCR reactions, 200ng in 2ul of sample per well were mixed with 5ul of TaqMan Gene expression MasterMix (ThermoFisher, Cat. # 4369016) and 2ul of H2O. The mix also contained 0.5ul of the following probes: Pdgfb (ThermoFisher, Assay ID: Mm00440677_m1) and housekeeping gene β-actin-FAM (ThermoFisher, Assay ID: Mm02619580_g1). No-RT controls were run side-by-side with samples. PCR was run using a Thermocycler programmed as follows: (1) 50°C for 2 minutes, (2) 95°C for 10 min, (3) 95°C for 15 secs, (4) 60°C for 1 minute. Steps 3 and 4 were repeated 40 times. Threshold cycles (Ct values) were used to calculate Pdgfb mRNA fold change. Briefly, ΔCt values were calculated by subtracting Pdgfb-Ct - β-actin Ct for each sample. ΔΔCt values were calculated by subtracting ΔCt values for each sample, to the average of ΔCt values of the control group for each experiment (Vehicle or BL OFF). Fold change was calculated with the following formula: 2ˆ(-ΔΔCt).

#### Western blot

Dorsal Root Ganglia (DRG) samples were homogenized in 200μl of modified RIPA IP Lysis buffer suitable for preservation of membrane proteins (cat. 87787, Thermo Fisher, 25 mM Tris-HCl pH 7.4, 150mM NaCl, 1 mM EDTA, 1% NP-40 and 5% glycerol), supplemented with Protease inhibitor cocktail (Sigma, Cat. 13786 1:500), Phosphatase inhibitor cocktail 2 (Sigma, Cat. P5726 1:200), and Phosphatase inhibitor cocktail 3 (Sigma, Cat. P0044 1: 200). After sonication (3 x 5 seconds) lysates were centrifuged (13,000*g*) and pellet was discarded. Protein estimation was performed using a BCA assay (cat. A55864, ThermoFisher). For the western blot procedure, samples were adjusted to 23g of protein in 4X Laemmli Sample Buffer (Biorad, cat.1610747) and RIPA IP buffer. Proteins were run in a 6%, Tris-Glycine, 1mm, Mini Protein Gels 15 wells (ThermoFisher, cat.XP00065BOX), using NuPage Mes SDS Running Buffer (ThermoFisher, cat.NP0002) at 100V for 90 minutes. Samples were run alongside 10μL ladder, PageRuler™ Plus Prestained Protein Ladder (ThermoFisher, cat.26619). Proteins were transferred onto a nitrocellulose membrane by wet transfer method. Membrane was blocked for 1 hour at room temperature in blocking buffer: 5% BSA dissolved in 0.01% TBS-T buffer. Then, membranes were incubated overnight at 4C with antibodies dissolved in blocking buffer: HRP conjugated anti β-actin mAb, (Sigma, cat. A3854, 1:20,000), rabbit anti PDGFRβ (ThermoFisher, cat. MA5-15143, 1:1,000), rabbit anti-PDGFRβ Y1021 (Bioss, cat. bs-3322R-TR, 1:1000). After washing, membranes were incubated with secondary antibody for 1 hour at room temperature in blocking buffer: Goat anti-rabbit – HRP conjugated (Cell Signaling, cat. 7074S, 1:5,000). Chemiluminescence signal was revealed using SupraSignal West Pico PLUS substrate (ThermoFisher, cat. 34580).

Analysis of bands was done using ImageJ Fiji software (NIH, Bethesda, USA). PDGFRβ is detected in bands spanning across 124kDa (predicted based on sequence) and 180kDa (full glycosylated protein). Area between 124kDA and 180kDa was delineated and quantified. Signal intensity was normalized to signal intensity of β-actin band detected at 42kDa.

#### Fluorescence *in situ* hybridization

Skin tissue was transferred from 2% PFA to 30% sucrose for 24hrs, and then to 20% sucrose until sunk and stored at 4°C. Tissue was snap frozen in OCT, 14μm sections were obtained using a cryostat at -18°C and mounted on glass slides (FisherBrand™ Superfrost™ Plus Microscope Slides: 1255015). Sectioned tissue was stored at -80°C for at least 24 hours before use.

Slides stored at -80°C were transferred to an oven and baked at 60°C for 1 hour before post-fixation in 4% paraformaldehyde (PFA) for 1 hour at 4°C. After 2 washes in distilled water, slides were dehydrated in 50%, 70%, and 100% ethanol each for 5 mins. A hydrophobic pen was used to draw a barrier around tissue sections prior to incubation in target retrieval solution (ACDBio) at 95°C for 5 mins. Slides were then processed using the RNAscope kit V2 (ACDBio, Cat. 323100, Biotechne). Slides were incubated in hydrogen peroxide 10 min at RT and protease III at 40°C for 30 mins. Probes targeting mRNAs sequences of the platelet-derived growth factor type b (Pdgfb, Mm-PDGF-B-C1 (ACD Bio Cat. 42451)), platelet-derived growth factor receptor beta (Pdgfrβ, Mm-PDGFr-B-C2 (ACD Bio Cat. 411381-C2)) and opioid receptor mu-1 (Oprm1, Mm-Oprm1-C3 (ACD Bio Cat. 315841-C3)), were added to the slides and incubated at 40°C for 2hrs. Probe signals were then amplified using manufacturer’s AMP solutions 1, 2, and 3 via incubation at 40°C for 30 mins, 30 mins, and 15 mins, respectively. Probes were assigned a TSA Plus fluorophore (TSA Vivid 520 - Pdgfb, TSA vivd 570 - Pdgfrβ, TSA vivid 650 - Oprm1) and incubated for 30 min at 40°C. Slides were incubated with HRP blockers at 40°C for 15 mins following each TSA Plus fluorophore incubation. All tissue was stained with DAPI (ACD Bio Cat. 323108), coverslipped and slides stored at 4°C until imaging.

#### RNAscope imaging and analysis

Skin tissue labelled with fluorescent mRNA probes was imaged using a BZ-8000 widefield fluorescence microscope (Keyence) using a 40X objective lens. Z-stacks were acquired (3μm total, 1μm steps). Two ROIs were imaged per tissue sample per mouse and Maximum Intensity projections were used for analysis.

All images were uploaded to QuPath for mRNA spots analysis. Cell segmentation was performed based on DAPI nuclear staining. The basal layer of keratinocytes was segmented using the following settings: 10μm background radius, Sigma 0.75, Minimum area: 2μm2, Maximum Area 400μm2, Cell expansion: 2μm. The upper layers of keratinocytes were segmented using the following settings: 8μm background radius, Sigma 0.75, Minimum area: 2μm2, Maximum Area 400μm2, Cell expansion: 4μm. Thresholds for detection of spots (subcellular detections, mRNA copies) were calculated as 35% of the maximum intensity of each channel in the ROI. Minimum and maximum spot size set at 0.3 and 2μm, respectively. Background noise was calculated by averaging the number of estimated spots per cell across all samples in an experiment. Cells were then considered positive for an mRNA if they contained at least the average detected spots. Cell populations were categorized as: (1) negative for both Pdgfb and Oprm1 mRNA (Pdgfb-/Oprm1-), negative for Pdgfb and positive for Oprm1 (Pdgfb-/Oprm1+), positive for Pdgfb and negative for Oprm1 (Pdgfb+/Oprm1-), and positive for Pdgfb and positive for Oprm1 (Pdgfb+/Oprm1+). Then, we calculated the average number of Pdgfb mRNA copies in all Oprm1+ cells per treatment group.

#### Primary cultures of keratinocytes

Glabrous skin pads were collected from mice on day 5 after vehicle or morphine i.pl. treatments. They were rinsed in betadine, Hank’s Balanced Salt Solution without calcium or magnesium (HBSS; from Fisher Scientific cat # 14170112), then 70% EtOH (<1 minute per rinse). Skin pads were placed in Dispase solution (8mg/ml Dispase II [neutral protease, grade II from Sigma Aldrich cat# 4942078001] in HBSS with 0.1% sterile Penicillin-Streptomycin [P/S; 5,000U/ml from Fisher Scientific cat#15070063] and 0.5μg/ml Amphotericin B solution fungizone [from Sigma Aldrich cat# A2942]) for 1 hour at 37°C. Skin pads were agitated every 10 minutes during this incubation. The epidermis was then peeled from the dermis of each pad, and the epidermis was submerged in Trypsin-EDTA (0.05%, from Fisher Scientific cat#25300054) at 37°C for 30 minutes, agitating every 5 minutes. Next, equal volume of HBSS was added to the Trypsin-EDTA solution and the skin pads were mechanically ground with a pipette tip for further dissociation. This solution was passed through a 70um cell strainer, then was centrifuged at 0.6RCFx1000 for 5 minutes. The top layer was removed, and the cells pelleted at the bottom were resuspended in complete Defined Keratinocyte Serum Free Basal Medium (KSFM [from Fisher Scientific cat #10744019], with 0.2% defined growth supplement [from Fisher Scientific cat #10744019], 0.1% P/S, 100μg/ml Epidermal growth factor [EGF; from Fisher Scientific cat#53003-018], and 0.5μg/ml Amphotericin B solution fungizone). Cells were plated on collagen (from Fisher Scientific cat#NC1558174)-coated coverslips and allowed to grow at 37°C and 5% CO2 with KSFM media changes every other day. Keratinocytes were recorded using whole-cell patch clamp 4-7 days after plating.

#### Electrophysiology

These experiments were done in primary cultures of keratinocytes, collected from mice either treated for five days with saline (5μl i.pl.) or with morphine (5μg/5μl i.pl.). The saline control accounts for the effect of repeated i.pl. injections in mice hindpaw and allows to specifically examine the effect of repeated peripheral morphine exposure on keratinocytes’ physiology. Whole-cell patch clamp recordings of keratinocytes were performed in a recording chamber filled with artificial cerebral spinal fluid (aCSF) of the following composition (mM): 126 NaCl, 2.5 KCl, 1.3 NaH_2_PO4•H_2_O, 1 MgCl_2_, 2 CaCl_2_, 26 NaHCO_3_, 10 D-Glucose, at a rate of 2-3 ml/min at room temperature. Briefly, borosilicate glass electrodes (1.5 mm OD, 7– 14 MΩ resistance) were filled with an internal solution containing (mM): 120 K-methanesulfonate; 20 KCl; 10 HEPES; 2 ATP, 1 GTP, and 12 phosphocreatine. Following seal rupture, series resistance was 29.2 ± 1.1 MΩ, fully compensated in current clamp recording mode, and periodically monitored throughout recording sessions. Recordings with changes of series resistance larger than 20% were rejected. Current-Voltage traces were recorded with the following parameters: 14 sweeps with increasing injected current of +50 pA with an initial injected current of -200 pA. Voltage and current traces in whole- cell patch-clamp were acquired with an EPC10 amplifier (HEKA Elektronik; Germany). Sampling was performed at 10 kHz and digitally filtered voltage, and current traces were acquired with PatchMaster 2.15 (HEKA Elektronik; Germany) at 2 kHz. All traces were subsequently analyzed off-line with FitMaster 2.15 (HEKA Electronik; Germany). In these experiments, three males and one female mouse per treatment group were used, 2-3 cells were recorded per animal. Mice Experimenter was blinded during patch experiment and analysis but was unblinded during data interpretation.

### Quantification and statistical analysis

All details on statistical analyses and results can be found in results section, figure legends, and in the supplemental tables provided for each figure. Behavioral and cell counting measures were performed by investigators blinded to experimental groups. All results are presented as mean ± s.e.m. Data were tested for normality and sphericity, and a Greenhouse Geisser correction was applied where appropriate. Assumption of Normality was tested either with the Shapiro–Wilk test or with a Q-Q plot. Student unpaired 2-tailed t-test was used to compare means of two independent groups. Repeated Measure two-way or three-way ANOVA were used with treatment and time or treatment, time and sex as variables, respectively. Tukey’s or Šídák's were used for post hoc comparisons of groups. GraphPad Prism 9.5 and RStudio and R software (R 4.3.1.) were used for data treatment and statistical analysis. All statistics are summarized in Tables S1, S2, S3, and S4. P<0.05 was considered as significant.

## Supplemental information

Document S1. Figures S1–S5

Table S1. Excel spreadsheet related to Figures 1 and S1 Statistical analyses for the data in Figures 1 and S1.

Table S2. Excel spreadsheet related to Figures 2 and S2 Statistical analyses for the data in Figures 2 and S2.

Table S3. Excel spreadsheet related to Figure 3 Statistical analyses for the data in Figure 3.

Table S4. Excel spreadsheet related to Figure 4 Statistical analyses for the data in Figure 4.

Table S5. Excel spreadsheet related to Figures 5 and S4 Statistical analyses for the data in Figures 5 and S4.

Table S6. Excel spreadsheet related to Figure 6 Statistical analyses for the data in Figure 6.

Table S7. Excel spreadsheet related to Figures 7 and S5 Statistical analyses for the data in Figures 7 and S5.
