# Supplementary material for: Keratinocytes and PDGF-B/PDGFRβ signaling modulate peripheral opioid tolerance
Source: iScience. 2026 Jun 30;29(7):116634. doi: 10.1016/j.isci.2026.116634 (PMC13378143; doi:10.1016/j.isci.2026.116634)
Supplement: Document S1. Figures S1–S5 [file mmc1.pdf]

iScience, Volume *29*

Supplemental information

Keratinocytes and PDGF-B/PDGFRβ signaling modulate peripheral opioid tolerance

Luca Posa, Angelique J. Buton, Anita M. Khasnavis, Sophia A. Miracle, Kathryn M. Albers, Matthew J. Fanelli, Timmy Le, Mackenzie Gamble, Ashley K. McDonald, Gilles Martin, Salome Fabri-Ruiz, Zachary Freyberg, Ryan W. Logan, and Stephanie Puig

SUPPLEMENTAL VIDEOS, FIGURES AND TABLES

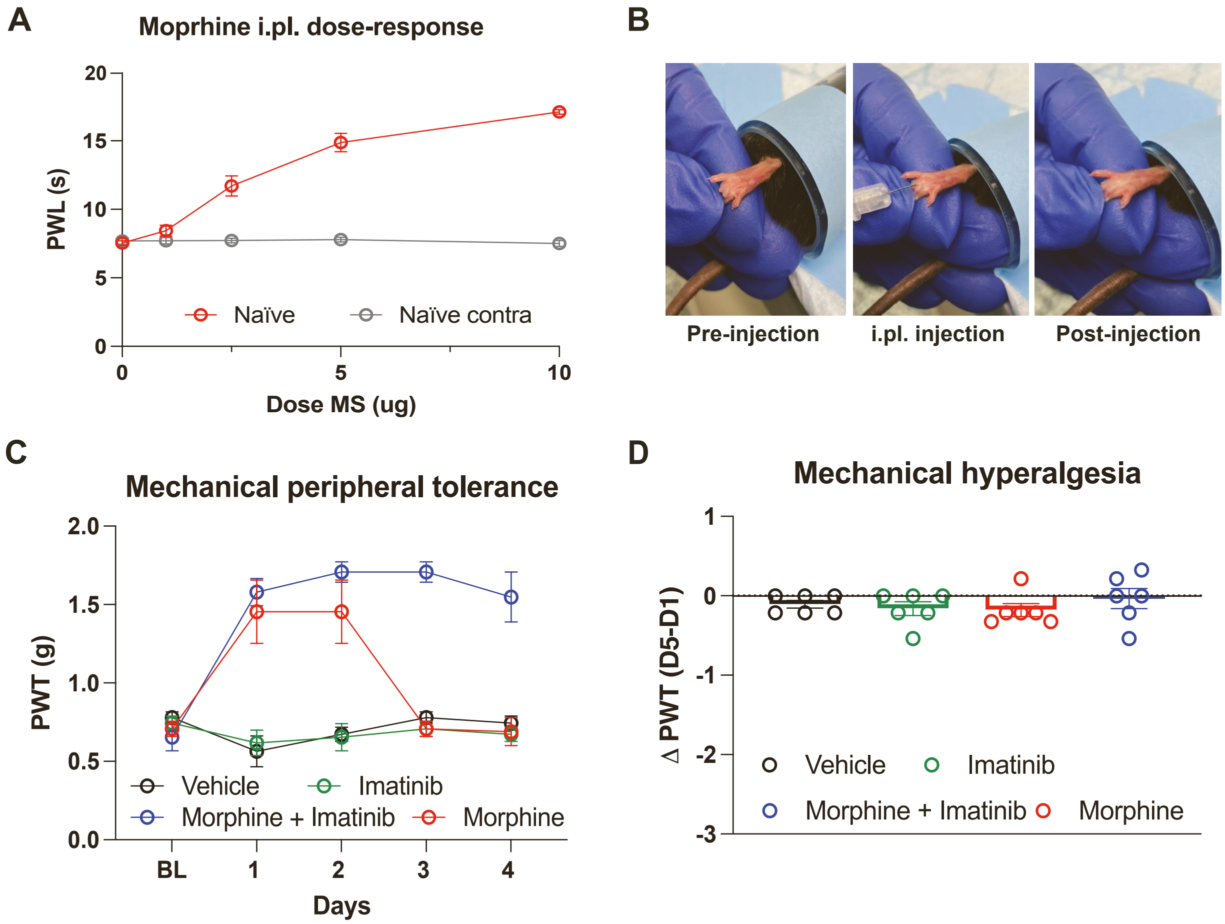

Figure S1. Peripheral morphine injections induce local analgesia without injury and imatinib blocks peripheral tolerance to mechanical stimulation. Related to Figure 1

(A) Dose-response of i.pl. injection of morphine showed ipsilateral acute antinociception in a dose-dependent fashion.

(B) Picture showing the i.pl. injection technique. Note, no bleeding or tissue damage is caused by the injection.

(C) Mechanical peripheral tolerance develops after repeated morphine i.pl. injections. Co-administration of morphine + imatinib prevents the decrease in paw withdrawal threshold (PWT) on days 3 and 4.

(D) Repeated i.pl. injections of morphine do not produce mechanical hypersensitivity in the ipsilateral paw.

BSL = baseline, D = day, OIH = opioid-induced hyperalgesia. N = 3 per group/sex. Two-way Repeated Measures ANOVA (**A, C**) or Ordinary One-way ANOVA (**D**). Data are expressed as mean ± s.e.m. Detailed statistics information can be found in **Table S1.**

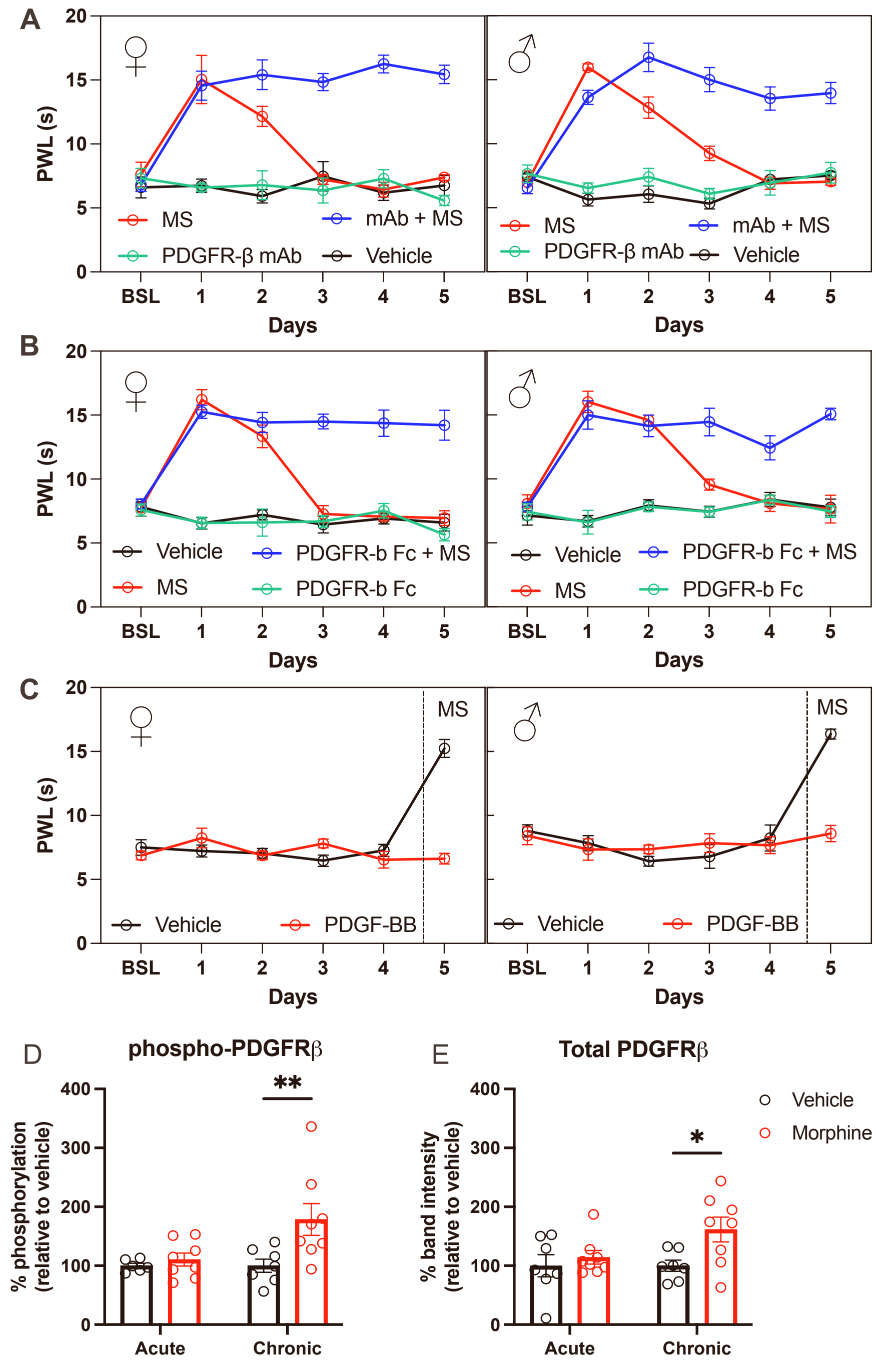

Figure S2. PDGFRβ signaling is both necessary and sufficient for peripheral tolerance in both sexes. Related to Figure 2.

(A) Morphine peripheral tolerance develops upon repeated morphine i.pl. injections. Co-injection of morphine + anti-PDGFRβ mAb blocks peripheral tolerance in females (**A left**) and males (**A right**) mice. N = 4-5/sex/treatment.

(B) Morphine peripheral tolerance develops upon repeated morphine i.pl. injections. Co-injection of morphine + PDGFRβ Fc blocks peripheral tolerance in females (**B left**) and males (**B right**) mice. N = 5-6/sex/treatment.

(C) Repeated i.pl. administration of recombinant PDGF-B blocks analgesia to a morphine challenge on day 5 in female (**C left**) and male (**C right**) mice. N = 5-6/sex/treatment.

(**A-C**) Two-way or three-way Repeated Measures ANOVA followed by Tukey's (**A-B**) or Šídák's (**C**) multiple comparisons test.

(D) Chronic (5 consecutive days) but not acute administration of morphine i.pl. induced phosphorylation of PDGFRβ on the tyrosine 857 (Y857) residue in DRGs. 2-way ANOVA followed by Uncorrected Fisher LSD. N = 7-8 (3-4/sex/group). *p<0.05 vs chronic vehicle.

(E) Chronic (5 consecutive days) but not acute administration of morphine i.pl. induced increase in total PDGFRβ expression in DRGs. 2-way ANOVA followed by Uncorrected Fisher LSD. N = 8 (4/sex/group). *p<0.05 vs chronic vehicle.

For all figures: BSL = baseline, MS = Morphine Sulfate, Data are expressed as mean ± s.e.m. Detailed statistics information can be found in **Table S2.**

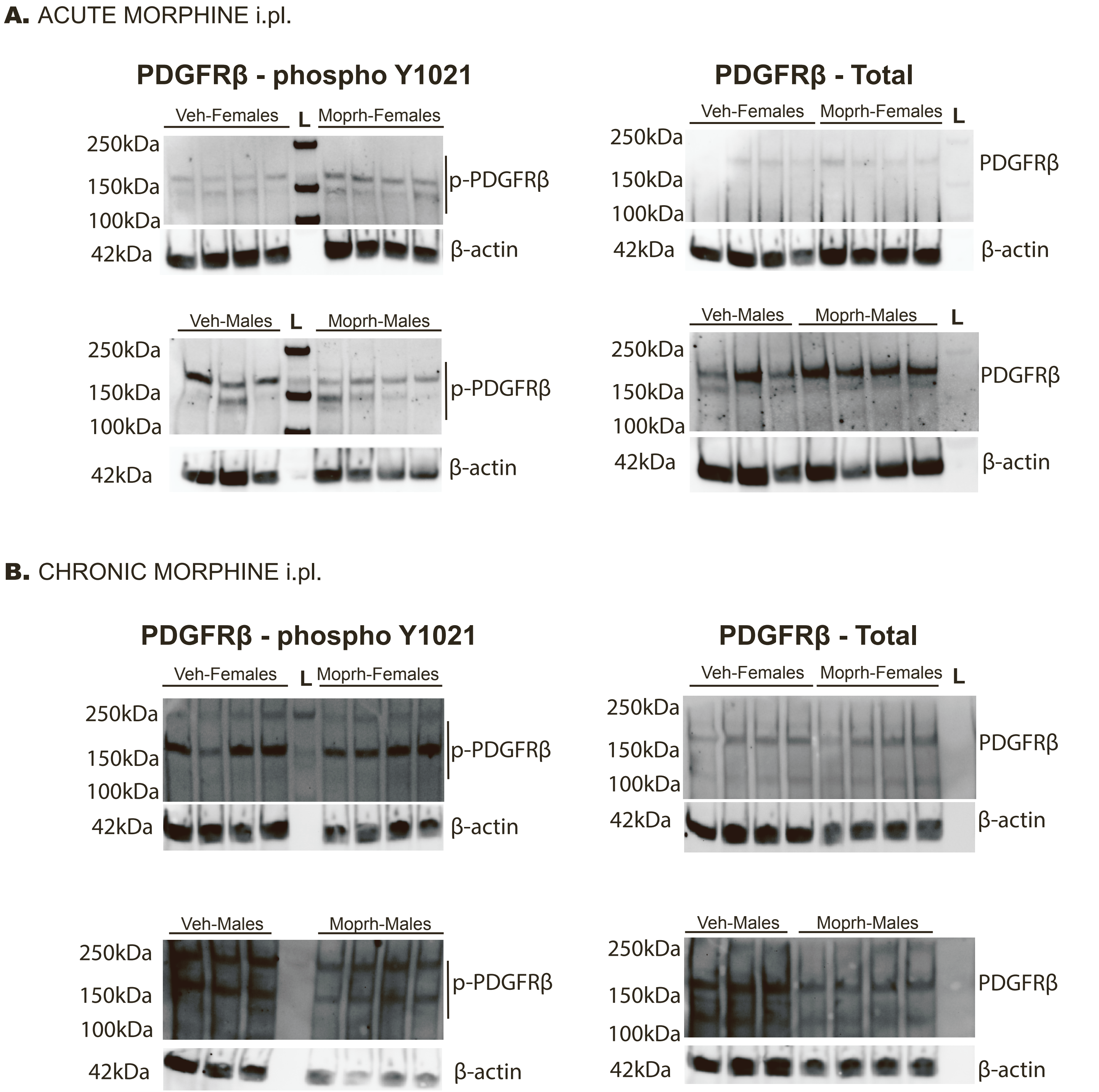

Figure S3. Western blots of DRGs collected after acute and repeated morphine i.pl. injections. Related to Figure 2 and S2

(A) Western blot images of DRG samples collected from males and females injected with vehicle or morphine i.pl. and collected 20min after the injection. Western blots were probed with antibodies detecting phosphor-PDGFRβ (**left**), total PDGFRβ (**right**) and β-actin as loading control for normalization to protein amount per well.

(B) Western blot images of DRG samples collected from males and females injected with vehicle or morphine i.pl. for five consecutive days and collected 20min the injection on day five. Western blots were probed with antibodies detecting phosphor-PDGFRβ (**left**), total PDGFRβ (**right**) and β-actin as loading control for normalization to protein amount per well. For all figures: L = Ladder, Morph = Morphine. Data was quantified and is represented in **Figure S2.**

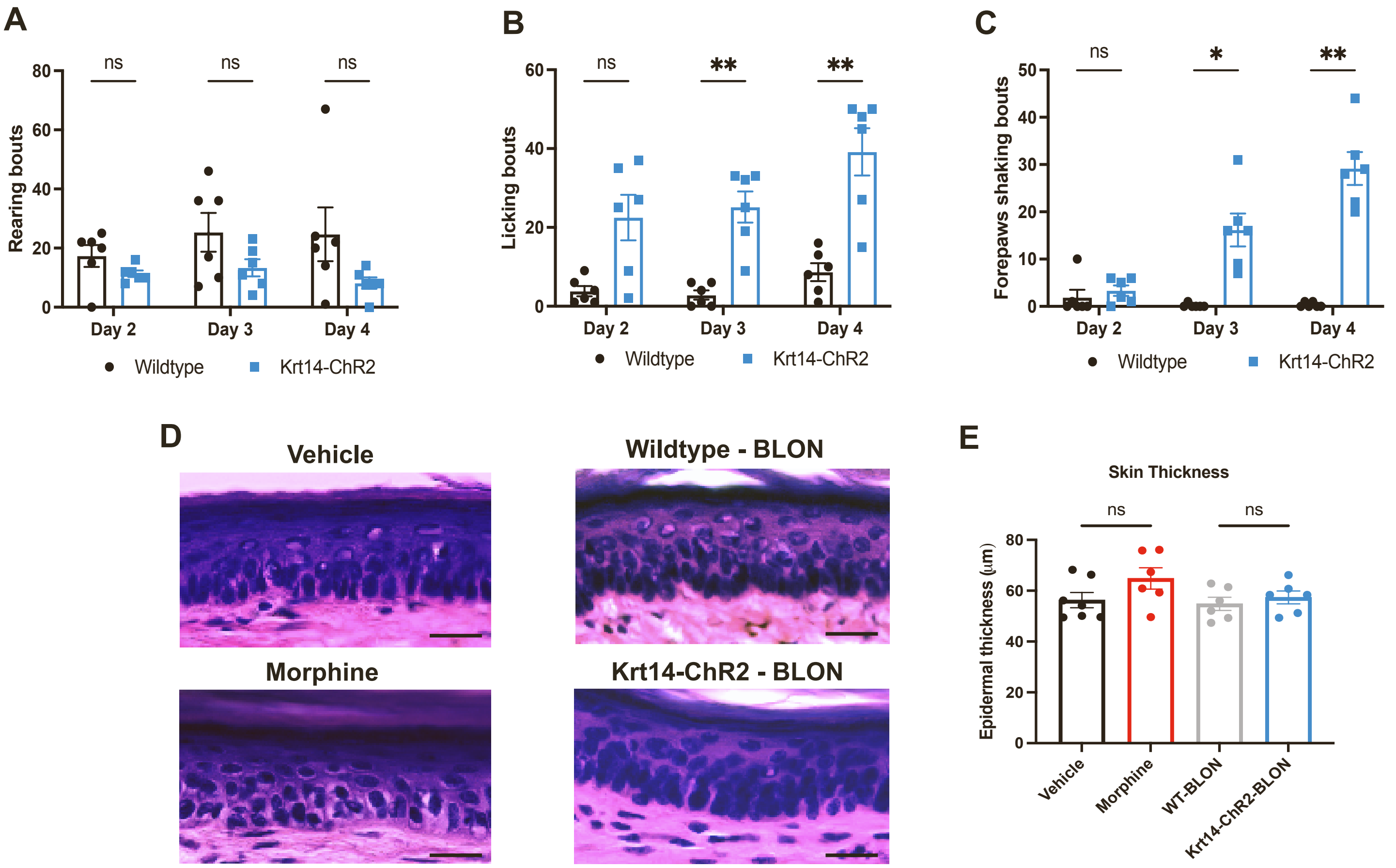

Figure S4. Behavioral and histological characterization of Krt14-ChR2 exposure to blue-light. Related to Figure 5.
(A) Krt14-ChR2 and WT littermates display similar total rearing bouts during Blue-Light exposure. N = 6 mice/group. See also **Video S1** for supplemental information on impact of blue light exposure.
(B) Krt14-ChR2 show an increase in licking bouts compared to WT littermates during Blue-Light exposure. N = 6 mice/group.
(C) Krt14-ChR2 display an increase in forepaw shaking bouts compared to WT littermates during Blue-Light exposure. N = 6 mice/group.
(D) Representative images of H&E staining of mouse skin showing no change in overall skin anatomy after 5 days of Blue-Light exposure. Scale bar = 10µm
(E) Krt14-ChR2 and WT littermates show similar epidermal thickness after 5 days of Blue-Light exposure. N = 5-7 mouse/group.
For all panels: Two-way ANOVA followed by Tukey's multiple comparisons test. $^*p<0.05$, $^{**}p<0.01$. Data are expressed as mean ± s.e.m. Detailed statistics information can be found in **Table S5**.

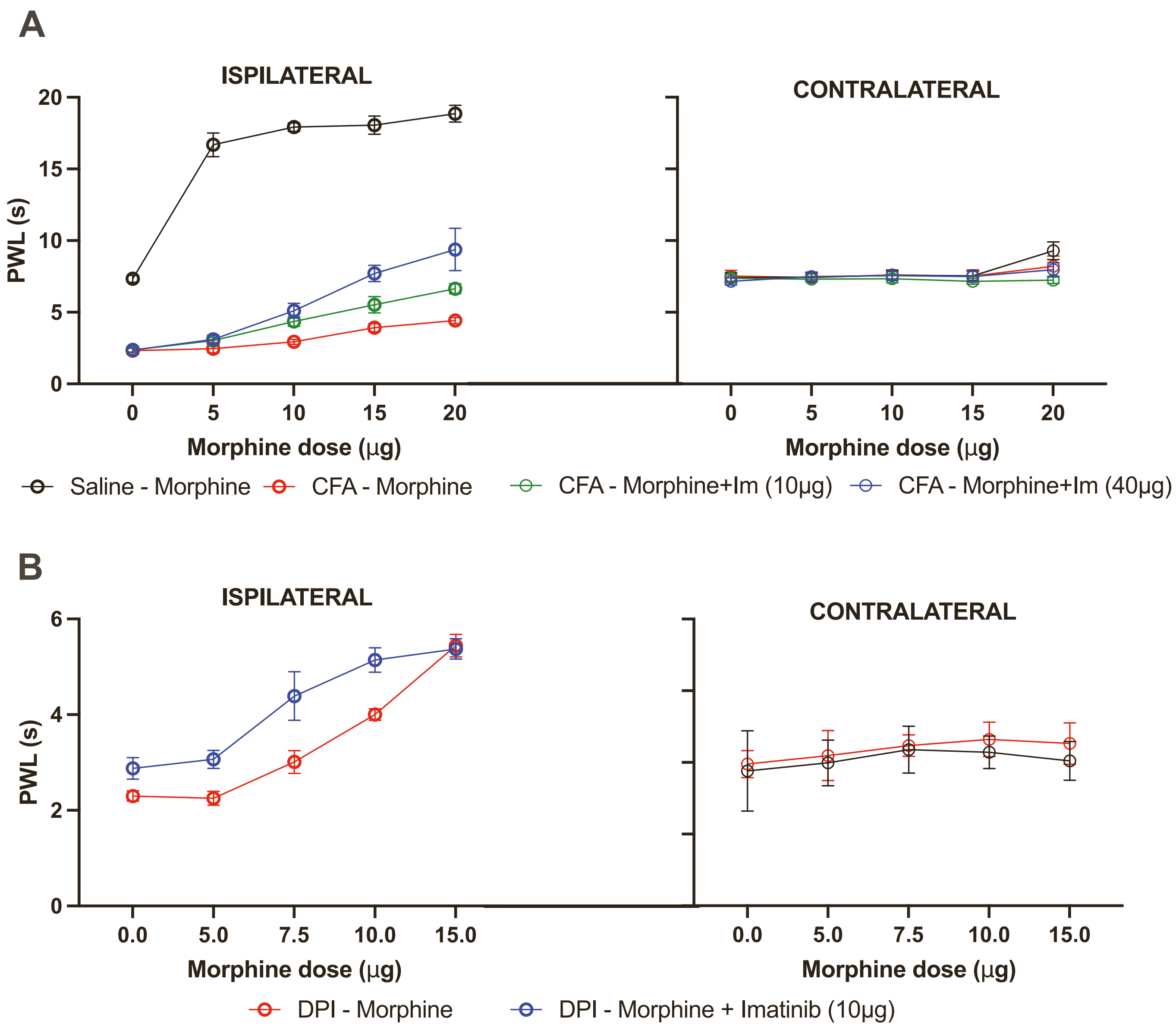

Figure S5. Morphine i.pl. dose response study in CFA and DPI pain models. Related to Figure 7.
(A) Dose-response curve of morphine +/- imatinib in the CFA model in the ipsilateral and the contralateral paw.
(B) Effect of i.pl. imatinib on peripheral morphine tolerance. Imatinib at 10 µg (i.pl.) dose not block peripheral morphine tolerance.
(C) Effect of i.pl. imatinib on peripheral morphine tolerance. Imatinib at 40 µg (i.pl.) effectively prevents the development of peripheral morphine tolerance. Moreover, co-administration of morphine and imatinib reverses peripheral tolerance in mice previously tolerant to morphine.
For all figures: N = 3/mice/sex/group. Two-way ANOVAs. Data are expressed as mean ± s.e.m. Detailed statistics information can be found in **Table S7**.
